# Molecular identification of the wheat male fertility gene *Ms1* and its prospects for hybrid breeding

**DOI:** 10.1038/s41467-017-00945-2

**Published:** 2017-10-11

**Authors:** Elise J. Tucker, Ute Baumann, Allan Kouidri, Radoslaw Suchecki, Mathieu Baes, Melissa Garcia, Takashi Okada, Chongmei Dong, Yongzhong Wu, Ajay Sandhu, Manjit Singh, Peter Langridge, Petra Wolters, Marc C. Albertsen, A. Mark Cigan, Ryan Whitford

**Affiliations:** 1 Australian Centre for Plant Functional Genomics, School of Agriculture, Food & Wine, University of Adelaide, Waite Campus, Urrbrae, SA 5064 Australia; 2 Plant Breeding Institute, University of Sydney, PMB 4011, Narellan,, NSW 2567 Australia; 3 DuPont Pioneer Hi-Bred International Inc., 7250 NW 62nd Avenue, Johnston, IA 50131-0552, USA

## Abstract

The current rate of yield gain in crops is insufficient to meet the predicted demands. Capturing the yield boost from heterosis is one of the few technologies that offers rapid gain. Hybrids are widely used for cereals, maize and rice, but it has been a challenge to develop a viable hybrid system for bread wheat due to the wheat genome complexity, which is both large and hexaploid. Wheat is our most widely grown crop providing 20% of the calories for humans. Here, we describe the identification of *Ms1*, a gene proposed for use in large-scale, low-cost production of male-sterile (*ms*) female lines necessary for hybrid wheat seed production. We show that *Ms1* completely restores fertility to *ms1d*, and encodes a glycosylphosphatidylinositol-anchored lipid transfer protein, necessary for pollen exine development. This represents a key step towards developing a robust hybridization platform in wheat.

## Abstract (web summary)

Heterosis can rapidly boost yield in crop species but development of hybrid-breeding systems for bread wheat remains a challenge. Here, Tucker *et al*. describe the molecular identification of the wheat *Ms1* gene and discuss its potential for large-scale hybrid seed production in wheat.

## Introduction

With the predicted growth in world population to over nine billion by 2050, the Food and Agriculture Organization of the United Nations (July 2005) set a target of 60% increased food production by that year. This is an ambitious target for two reasons: there are serious concerns about the viability of existing production systems and the sustainability of current growth rates in crop production, and the predicted environmental changes are expected to have an overall negative effect on agricultural production, with serious crop declines in some countries. Wheat is grown more widely than any other crop and delivers around 20% of our food calories and protein^1^. To increase global production by 60% will require a lift in the rates of gain from the current 1 to 1.6% per annum. Improvements in disease resistance and stress tolerance offer opportunities for small increases in productivity but major jumps in yield are hard to achieve and are expected to come through shifts in the way we breed wheat and other crops. However, many important new genetic and genomic technologies are difficult to apply to wheat since this plant has a large complex genome, an allohexaploid, which is 50 times larger than rice.

One of the most promising options for achieving significant boosts in yield across diverse production environments is through hybrid breeding. Hybrids offer two important advantages: first, heterotic yield gains of well over 10%, and improved yield stability have been reported^2–4^, and second, hybrid seed production would act as a major stimulant for investment in wheat improvement from both the public and private sectors. However, the competitiveness of wheat hybrids relative to line varieties will depend on hybrid seed production costs^5^.

Lowering hybrid seed production costs depends on a reliable and inexpensive system that forces outcrossing. Wheat male sterility and restoration systems were first developed in the 1960s, but many of them were proved to be impractical and deemed commercially high risk^6^. Relative to systems based on chemical hybridizing agents and cytoplasmic male sterility, the use of non-conditional nuclear-encoded recessive male steriles (*ms*) would offer major advantages for hybrid breeding. The value of recessive male steriles was first recognised in 1972 with the proposal of the XYZ system^7^. This system aimed to overcome the costs associated with propagating pure stands of male steriles by cytogenetic chromosomal manipulation^7, 8^. A further advantage of recessive male steriles came through the opportunity to broaden parental line choice, avoid negative alloplasmic and cytoplasmic yield penalties, as well as alleviate the problems associated with incomplete fertility restoration. A cost-effective and flexible hybridization platform that uses a recessive male sterile is Seed Production Technology (SPT)^9^ developed for maize and rice hybrid seed production (Supplementary Fig. 1). This platform overcomes many of the problems with large-scale production of male steriles for use as female parents in hybrid breeding. SPT uses a maintainer line solely for the propagation of non-GM homozygous recessive male-sterile parents; therefore, F_1_ hybrids provided to farmers are considered to be non-GM.

Developing an equivalent platform for hybrid wheat breeding requires the identification of a suitable non-conditional, nuclear-encoded recessive male sterile. These types of mutants are particularly rare and difficult to detect in polyploids due to genetic redundancy. Many of our major crops and food plants are polyploids, including wheat, oats, potato, sweet potato, peanut, sugarcane, cotton, kiwifruit, strawberry, and plums. For example, only ten nuclear-encoded wheat male-sterile mutants have been identified to date^6^, in contrast to 108 mutants in diploid barley^10, 11^. Polyploidy not only makes it difficult to find suitable male-sterile mutations but also complicates deploying mutants since multiple mutations would be needed to deal with genetic redundancy^12^ and this increases breeding costs and population sizes needed for introgression of each additional mutation. The most cost-effective mutants would be single locus encoded. In wheat, only two of the ten mutant loci are reported to fit this criterion. These are *ms1* and *ms5* located on chromosomes 4BS and 3AL, respectively^13^.

The first *ms1* mutant was observed in Australia in the late 1950s^14^. This spontaneous mutant named *Pugsley’s* male sterile was followed by the identification of *Probus* and *Cornerstone* male steriles from an X-ray-induced wheat mutant population^15, 16^. Cytogenetic and linkage analysis showed these to be allelic and they were designated as *ms1a*, *ms1b* and *ms1c*, respectively^15, 17–19^. In 1976, additional monogenic recessive male steriles were identified from an ethyl methanesulfonate (EMS)-treated population^20^. Three mutants were allelic to *ms1*, and designated as *ms1d*, *ms1e* and *ms1f* ^13, 21^ while the fourth mutant was nonallelic to *ms1* and designated as *ms5* ^13^. However, even for *ms1*, the variability between backgrounds and mutant alleles, and problems with male sterility penetrance were reported^22–24^. In order to address these problems, it is necessary to identify the gene underlying the *Ms1* locus and explain its function.

Here, we describe the identification of the *Ms1* gene sequence (*TaMs1*) by map-based cloning and demonstrate its function in male fertility by complementation of the *ms1d* mutant. *TaMs1* encodes a glycosylphosphatidylinositol (GPI)-anchored lipid transfer protein, which is necessary for pollen exine development. The identification of the *Ms1* gene sequence represents a key step towards developing a robust hybridization platform in wheat similar to the maize SPT.

## Results

### *Ms1* encodes a GPI-anchored lipid transfer protein

**Fig. 1:**
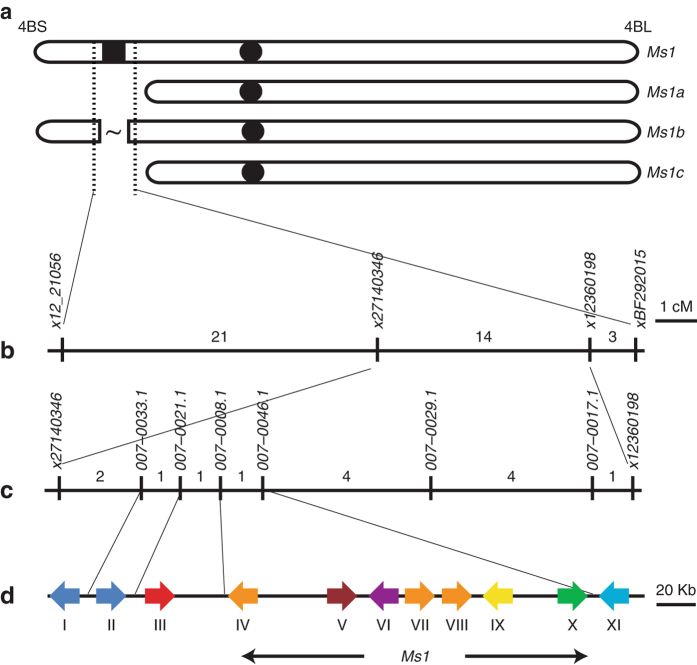
Map-based cloning of the *Male sterility 1* locus on chromosome 4BS. *Ms1* was initially mapped to the interval between × *12_21056* and *xBF292015* based on genotyping a (**a**) deletion mutant allele series, and then to an (**b**)  ~ 0.5 cM interval between ×*27140346* and ×*12360198* based on 7000 F_2_ segregants. **c** Fine mapping using 14 recombinants deliminated *Ms1* to a (**d**) 251-Kb genomic region in wheat. Marker names are in italics. The numbers indicate recombinants identified for each marker interval. Coloured arrows I to XI denote the position and orientation of predicted wheat genes with a similarity to *Brachypodium* genes Bradi1g13040 (Cupin domain-containing protein), Bradi1g13040 (Cupin domain-containing protein), Bradi2g05445 (60S ribosomal protein), Bradi1g13030 (Lipid Transfer Protein-Like 94), Bradi4g44760 (F-box/LRR-repeat protein 3), Bradi1g69240 (U-box domain-containing protein), Bradi1g13000 (Lipid Transfer Protein-Like 72), Bradi1g12990 (Lipid Transfer Protein-Like 71), Bradi1g12980 (Putative Parafibromin), Bradi1g12970 (Putative GNAT family acetyltransferase) and Bradi1g12960 (DUF581 domain-containing protein), respectively. The sequence is available via NCBI GenBank accession code KX447407

We followed a map-based cloning approach to isolate the *Ms1* gene sequence (chr. 4BS). Using syntenic regions on chromosomes 1, 3 and 4 from rice, *Brachypodium* and barley, respectively, we generated markers and tested their presence or absence in male-sterile mutants *ms1a*, *ms1b* and *ms1c*. The results revealed that *ms1a* and *ms1c* are terminal deletions while *ms1b* is an interstitial deletion of the chromosome 4BS covering approximately 14 centimorgans (cM) (Fig. 1). *Ms1*-flanking markers were identified by their presence in *ms1b* and their absence from *ms1c*. Using a population representing 7000 meioses and segregating for *ms1d*, we delimited the *Ms1* locus to a 0.5 cM interval between markers ×27140346 and ×12360198. Probes designed within the region bounded by these markers, were used to isolate and sequence BACs from durum and hexaploid wheat. Marker development from BAC-derived sequences and analysis of 14 recombinants across the region, further delimited *Ms1* between markers 007.033.1 and 007.0046.1. The mapped 251- Kb interval contains eight intact genes and one likely pseudogene (Supplementary Table 1).

RNAseq-based expression profiling identified one of these eight genes to be preferentially expressed in floral tissues (Supplementary Table 1). This gene (*TaMs1*) is predicted to encode a 219 amino acid polypeptide with a similarity to a large family of GPI-anchored lipid transfer proteins (LTPGs), for which it is a member of a Poaceae-specific clade (Supplementary Fig. 2). This gene was confirmed as *Ms1* through in planta complementation of the *ms1d* mutation and identification of the causative lesions in *ms1d*, *ms1e*, *ms1f* and a newly identified TILLING mutant (described below).

### *TaMs1* is necessary for pollen exine integrity

**Fig. 2:**
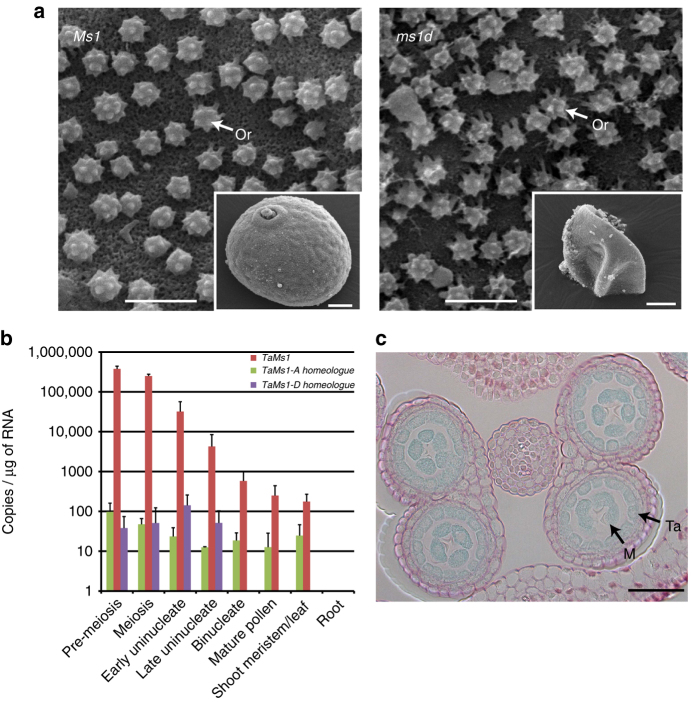
*TaMs1* confers male fertility and is expressed in developing wheat anthers. **a** Scanning electron micrographs showing the defects in tapetal cell surface-localised orbicule (Or) structures and pollen coat (inset) within male sterile (*ms1d*) vs. wild-type anthers (*Ms1*). Scale bars: 2 µm (inset 10 µm). **b** *TaMs1* and homeologue mRNA levels as detected by qRT-PCR in premeiotic to mature pollen-containing anthers, leaf/shoot apical meristem and roots. The data are means ± s.e.m (n = 3 biological replicates). **c** Histochemical GUS analysis of wheat anthers expressing a translational GUS fusion with *TaMs1* (native promoter). Transverse section of a wheat anther containing microspores undergoing meiosis showing cell-type-specific GUS expression. Ruthenium red-stained cell walls (pink). Scale bar: 50 µm. (Ta Tapetal cell, M Microspore)

*Arabidopsis* harbours over 20 LTPG genes for which only two of them have been characterised^25, 26^. *AtLTPG1* and *AtLTPG2* are required for cuticular wax accumulation or for export onto stem and silique surfaces. Epicuticular wax has lipid precursors common to sporopollenin, the major constituent of pollen exine, which is produced in sporophytic tapetal cells and transported to developing microspores in structures called orbicules. The analysis of *ms1* anthers revealed a disrupted orbicule and a pollen exine structure (Fig. 2a), which was first observed in early uninucleate microspores and typified by ectopic exine deposition and reduced electron-dense materials at the tapetal cell surface (Supplementary Figs. 3 and 4). No differences in the surface cuticle layer were observed between *ms1* and wild-type anthers (Supplementary Fig. 5). Furthermore, metabolomic profiling revealed that *ms1* anthers accumulate lipid monomers of sporopollenin (C16 and C18 long-chain fatty acids) relative to the wild type (Supplementary Fig. 6). Taken together, this suggests that *Ms1* is necessary for sporopollenin biosynthesis or transport. Transcriptional β-glucuronidase fusions and homeologue-specific qRT-PCR revealed only the B-genome-derived *TaMs1* is to be expressed during early microspore development (Fig. 2b, c).

### *TaMs1* exhibits functional divergence from its homeologues

Since no obvious differences in *TaMs1* coding potential were detected between homeoloci, the basis for *TaMs1*’s sub-functionalization between homeoloci is likely to be due to variation in transcription. We suggest that functional homeoalleles may still exist in the cultivated germplasm pool and this could account for the reports of poor sterility penetrance dependent upon the genotype and the mutant allele^22–24^. In each of these cases, the loss of *Ms1* is either via a large deletion (*ms1a* and *ms1c*) or chromosome arm (DT4BL) replacement; therefore, restoration of fertility is unlikely to be a consequence of the B-genome-derived *TaMs1*. By performing a *TaMs1* homeologue-specific qRT-PCR on anthers isolated from a partially fertile homozygote for *ms1c*, we attempted to answer the question on whether *TaMs1* homeoalleles can transcriptionally compensate for the loss of *TaMs1*-B. However, this does not seem to be the case since *TaMs1* and homeologous transcripts from cv. *Cornerstone* were all below the detection limits (Supplementary Fig. 7). It is, therefore, possible that other genomic loci are associated with fertility restoration in lines carrying *ms1* deletions.

**Fig. 3:**
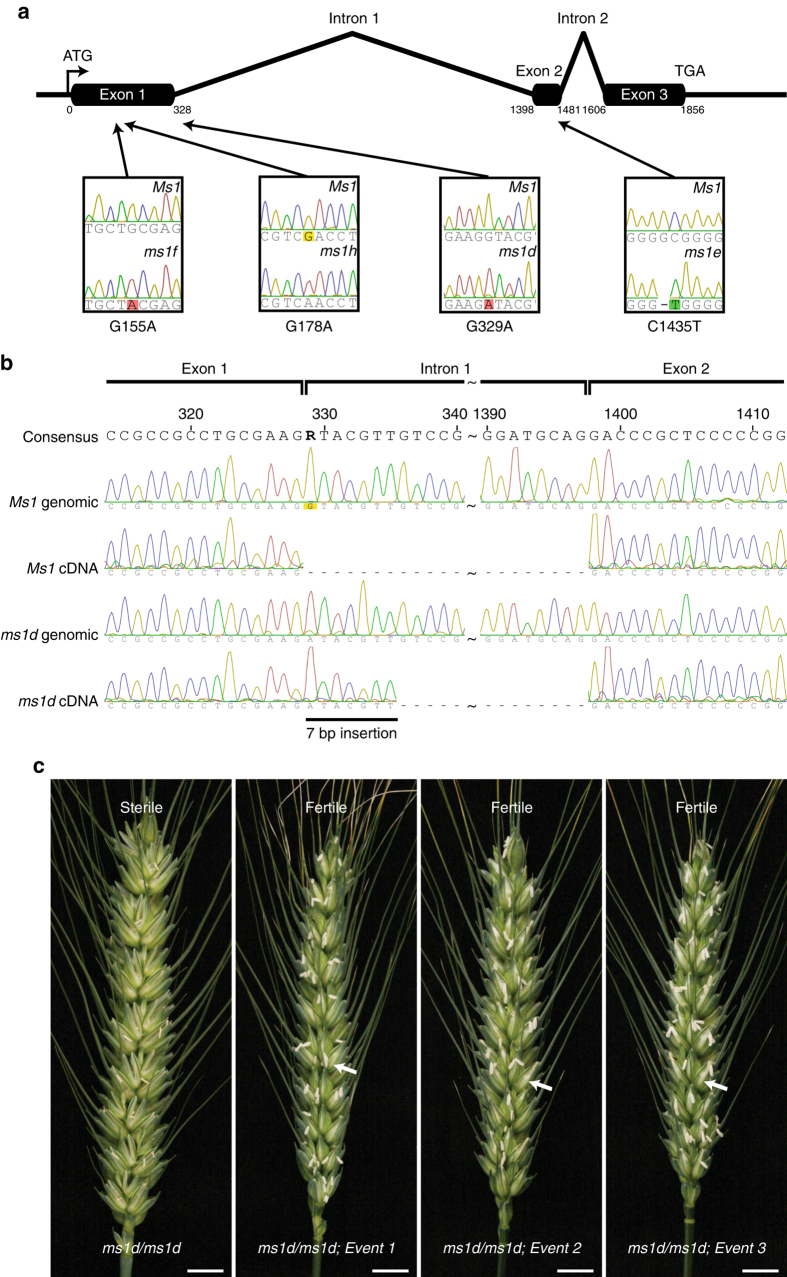
Identity of male sterility inducing lesions within *TaMs1* and in vivo complementation of *ms1d*/*ms1d* by *TaMs1*. **a** Schematic representation of the *TaMs1* (the three exons are shown as black boxes) gene depicting the relative positions (indicated by solid lines with arrowheads) of EMS-derived lesions (chromatogram insets) for *ms1d*, *ms1e*, *ms1f* and *ms1h*. Polymorphisms cause either a frame shift (*ms1d*, *ms1e*) or an amino acid transition in a conserved residue of TaMs1 (*ms1f*, *ms1h*). **b** Sequence chromatogram comparison between both mutant (*ms1d*) and wild-type genomic cDNAs. Polymorphism G329A of *ms1d* induces the use of a cryptic splice site (highlighted by a 7 bp insertion) within *TaMs1*. **c** Stable complementation of the *ms1d* mutant by *TaMs1*. Mature inflorescences of male-sterile *ms1d*/*ms1d*, and three independent transformants (Events 1–3), each homozygous for *ms1d*, and showing a self-seed set (arrow). Scale bars: 1 cm

The variable penetrance of *ms1* large-deletion mutants led us to investigate the utility of the available *Ms1* mutant alleles derived from an EMS-treated population^27^. Chromosome 4BS-specific full-length coding sequences from wild-type (*Ms1*) fertiles and the EMS-derived *ms1d*, *ms1e* and *ms1f* steriles were isolated and sequenced. A comparison of *Ms1* to *ms1*-derived sequences identified unique single-nucleotide transitions for each mutant allele (Fig. 3a). Transition G329A is unique to *ms1d* and unlikely to be a natural allelic variant, considering that the wild-type G is detected in all 192 spring wheat varieties tested (Supplementary Table 2). This SNP, at the first *Ms1* exon–intron boundary induces a cryptic splice site in the first intron, resulting in a coding sequence frame shift (Fig. 3b). *ms1e* transition C1435T is coupled with a 1 bp deletion in the second exon, resulting in a frame shift prior to the predicted C-terminal GPI anchor domain. *ms1f* transition G155A changes a highly conserved cysteine residue to a tyrosine (C52Y). This site is one of the eight cysteine motifs characteristic of LTP domains that appear to be important for the structural scaffold necessary for lipid binding^28^. In a parallel approach, we identified a G178A transition (designated as *ms1h*) in a TILLING screen of a soft wheat cultivar *QAL2000*, that induced male sterility similar to other mutant alleles (Supplementary Fig. 8) when it was in the homozygous condition (Fig. 3a)^29^. This mutation changes an aspartic acid to an asparagine (D60N) within the conserved LTP domain. Taken together, these findings indicate that both the GPI anchor and putative lipid-binding domains are necessary for Ms1 functionality.

### *TaMs1* functionally complements *ms1d*

The SPT hybridization platform incorporates a maintainer line (Supplementary Fig. 1) capable of propagating non-GM nuclear male-sterile lines for use as female parents in hybrid production. The SPT maintainer line is a homozygous recessive male sterile transformed with a SPT construct containing (i) a complementary wild-type male fertility gene to restore fertility, (ii) an α-amylase gene to disrupt pollination and (iii) a seed colour marker gene. We demonstrated that the α-amylase gene and seed colour marker function in wheat (Supplementary Fig. 9). However, the remaining key component of the SPT hybridization platform requires the demonstration of complementation of male sterility to fully restore fertility^9^. Therefore, we tested the ability of this gene sequence to complement *ms1d*. A 4.4-Kb genomic fragment containing *Ms1* was synthesised (*TaMs1*) and introduced into the wheat cultivar *Gladius* segregating for *ms1d*. Eleven independent *Agrobacterium*-mediated T_0_ transformants were generated (Supplementary Table 3). Four SNP markers closely flanking the *Ms1* locus allowed the selection of T_0_ regenerants that were homozygous for *ms1d*, whilst a seed-screenable marker (*DsRed*) was used to confirm the presence of *TaMs1*. SNP and seed colour detection identified six homozygous *ms1d* T_0_ regenerants with the introduction of the *TaMs1* gene. Selfed seed set analysis showed that all six T_0_ regenerants were fully fertile (Fig. 3c, Supplementary Table 3). Seventeen T_1_ progenies for two independent T_0_ lines (Event 1 and Event 7) were assayed for both the copy number and zygosity of the introduced *TaMs1* (Supplementary Table 4). The results revealed that all progenies were homozygous for *ms1d* with either zero, one or two copies of the exogenous *TaMs1*. Those progenies with no detectable introduced *TaMs1* were male sterile whilst those containing either one or two copies of the *TaMs1* transgene were self-fertile. These findings demonstrate that *Ms1* can fully restore fertility to the homozygous *ms1d* mutant.

## Discussion

An ambitious target of a 60% increase in wheat production by 2050 has been set to meet the predicted demand for this crop (FAO). A viable hybrid seed production system could deliver a third or more of this gain and currently, it is difficult to see any other technology that could achieve an equivalent impact. Several components are needed for an efficient hybrid system, which include optimising hybrid gains through defining heterotic pools and improving pollen vectoring by transitioning from a self-pollinating flower structure to the one that facilitates outcrossing. Based on experience in other hybrid systems, these components can be generated through targeted selection. However, forcing outcrossing through a reliable and manageable male sterility system requires the development of systems that are able to cope with the complexity of the hexaploid wheat genome.

The use of recessive male sterility has been an attractive prospect for hybrid seed production in wheat since it was first proposed in the 1950s but translating the concept to reality has proved to be elusive. The conundrum has been to maintain the male-sterile lines for use as pollen acceptors in commercial hybrid seed production fields. For example, the cytogenetic 4E-*ms* system, which utilises the novel mutant *ms1g* allele and a fertility-restoring chromosome from *Agropyron elongatum* ssp. *ruthenicum* Beldie (4E)^30^ was initially reported to be successful in enabling the production of a male-sterile seed. However, reports have since indicated residual pollen transmissibility of chromosome 4E,^31^ resulting in a selfed seed set within the male-sterile stands and thus reducing the purity of the hybrid seed produced. Therefore, alternate approaches are being sought to enhance this system. Now, with the isolation of *Ms1* and characterisation of most known mutations at this locus, we finally have the tools needed to develop an efficient means of bulking a male-sterile female-inbred seed (*ms1*/*ms1*), as is the case for SPT in maize. This represents a major cost saving for hybrid seed production and can overcome the seed purity issues inherent to the 4E-*ms* system. Coupled with a functional α-amylase gene for wheat pollen disruption and a seed-selectable marker, the identity and ability of the *TaMs1* gene sequence to completely restore viable pollen production in *ms1* plants represents the last critical step towards developing SPT for wheat.

To our knowledge, this study is the first to report a role of a GPI-anchored LTP in pollen exine development^32^. Given the observation that *ms1d* anthers contain pollen deficient in exine structural integrity, and that metabolomic profiling revealed a significant accumulation in free C16 and C18 fatty acids, it is reasonable to expect this to be a consequence of disruption in sporopollenin precursor transport from sporophytic tapetal cells to developing microspores. The finding that anther epidermal wax layers were not disrupted in *ms1d* anthers also indicates that TaMs1’s role is specific to exine development. Further studies are needed to elucidate the subcellular localisation of TaMs1 and its direct involvement in lipid binding.

*TaMs1*’s identification now also raises the question on whether previously reported male-sterile mutants may represent additional mutant alleles of this gene or its homeologues. Apart from the dominant male-sterile mutants *Ms2* ^33, 34^ and *Ms4* ^35^, no other mutants have been reported on homeogroup four. Both *Ms2* and *Ms4* were reported to be located on chromosome 4DS. However, recent cloning and complementation of the *Ms2* gene sequence *WMS*, reveals that it does not encode a *TaMs1* homeologue, but represents a novel gene sequence expressed only in sterile anthers as a consequence of a transposon insertion^36, 37^. Further research is necessary to determine whether *Ms4* is a mutant of the *TaMs1*-D homeologous gene sequence.

From a traditional breeding perspective, the molecular identity of the *TaMs1* gene sequence now allows the development of germplasm-specific markers for fast-tracking *ms1* introgression into diverse female-inbred parental lines. Moreover, complementation studies demonstrate that *ms1* is unique and contrasts with other reported wheat *ms* mutant alleles^38^ in that *ms1* behaves as a single-mutant locus in hexaploid wheat and a single copy of *Ms1* restores fertility. Given the characterisation of the *ms1* alleles described here and the observed variation in penetrance of sterility between these different alleles, understanding the relationship of these mutations to pollen production, as well as optimising *ms1* as a system for the production of a hybrid seed is now possible through the adoption of advanced breeding technologies such as gene editing^39, 40^. Gene editing would enable the generation of novel *ms1* alleles and allow simultaneous testing in different isogenic wheat backgrounds (e.g. spring and winter or hard and soft wheat). Further, once highly penetrant *ms1* alleles are identified, rather than introgression through conventional backcrossing, this new variant allele could be rapidly introduced into the most elite genetics by directly editing *TaMs1*. Adoption of new breeding technologies is likely to be particularly valuable in translating the results presented here in wheat to other polyploids suffering from a similar paucity of mutants. Such new technologies are necessary for the development of hybridisation systems to exploit heterosis as a means to increase seed production as global population increases.

## Methods

### Plant and DNA materials

Bread wheat lines used for fertility phenotyping, molecular marker development and genetic mapping were cv. Chinese Spring, Chinese Spring-derived nullisomic tetrasomic stocks^41^ (N4AT4D, N4AT4B, N4BT4A, N4BT4D, N4DT4A and N4DT4B), cv. *Gladius*, cv. *Pugsley’s* male sterile (*ms1a*)^14^, cv. *Probus* (*ms1b*)^16^, cv. *Cornerstone* (*ms1c*)^18^, cv. *Chris* and *Chris*-EMS-mutagenised lines FS2 (*ms1d*), FS3 (*ms1e*) and FS24 (*ms1f*)^21^. All cultivars, breeders’ lines or DNA samples for marker screening were obtained from various Australian wheat-breeding programmes, Australian Wheat and Barley Molecular Marker Program or the Australian Winter Cereals Collection (AWCC). The BAC library was derived from the *Triticum turgidum* ssp. durum cv. Langdon^42^. The EMS-mutagenised population used for TILLING was derived from the soft bread wheat cv. *QAL2000* ^29^.

### Plant growth and phenotyping

Plants for genetic mapping, cytological examination, expression analysis, TILLING and transformation donor material were sown at 5–6 plants per 6 l (8-inches diameter) pot containing soil mix. The soil mix consisted of 75% (v v^−1^) Coco Peat, 25% (v v^−1^) nursery-cutting sand (sharp), 750 mg l^−1^ CaSO_4_.2H_2_O (gypsum) 750 mg l^−1^ Ca(H_2_PO_4_)_2_.H_2_O (superphosphate), 1.9 g l^−1^ FeSO_4_, 125 mg l^−1^ FeEDTA, 1.9 g l^−1^ Ca(NO_3_)_2_, 750 mg l^−1^ Scotts Micromax micronutrients and 2.5 g l^−1^ Osmocote Plus slow-release fertilizer (16:3:9) (Scotts Australia Pty. Ltd.). The pH was adjusted between 6.0 and 6.5 using two parts of agricultural lime to one part of hydrated lime. Potted plants were grown either in controlled environment growth rooms at 23 °C (day) and 16 °C (night) or similarly temperature-moderated glasshouses in which the photoperiod was extended using 400 W high-pressure sodium lamps in combination with metal halide lamps to 12 h over winter months.

Individual plants were assessed for self-fertility by placing and sealing a glassine bag over each head before anthesis. Between three and ten heads per plant were collected for seed counting. The two basal and two apical sspikelets per head were eliminated from analysis due to their incomplete development. The total seed set and numbers of florets were counted on a per-head basis. The percentage of fertility for each spike or plant was calculated as follows:

$${\rm{\%}\ {fertility}} = \frac{{{\rm{Total}}\,{\rm{number}}\,{\rm{of}}\,{\rm{seeds}}\,{\rm{per}}\,{\rm{spike}}}}{{{\rm{Total}}\,{\rm{number}}\,{\rm{of}}\,1^\circ ,\,2^\circ \,{\rm{and}}\,3^\circ \,{\rm{florets}}\,{\rm{per}}\,{\rm{spike}}}} \times 100$$

A plant was deemed to be self-fertile if the total calculated percentage of fertility was greater than 60% or was equivalent to a wild-type control.

Pollen viability was assessed for three isolated anthers per plant (n = 3) by either acetocarmine–glycerin or Lugol (1% I_3_K solution) staining. Dissected anthers were mounted on glass microscope slides and pollen grains (*n* > 500 per sample) counted for staining by visualising them on a Zeiss Axio Imager M2 optical microscope coupled with a CCD camera (The University of Adelaide microscopy). Stainable pollen is represented as the mean ± standard deviation for nine samples per genotype.

### Histochemical staining cytological examination

Anthers containing premeiotic microspores to mature pollen were isolated from wheat plants identified to contain the *TaMs1*::*gusplus* cassette. Histochemical GUS activity was detected using 5-bromo-4-chloro-3-indolyl-beta-D-glucuronic acid (Gold Biotechnology, Inc). The samples were incubated in a 1 mM X-Gluc solution in 100 mM sodium phosphate at pH 7.0, 10 mM sodium ethylenediaminetetraacetate, 2 mM FeK_3_(CN)_6_, 2 mM K_4_Fe(CN)_6_ and 0.1% Triton X-100. After vacuum infiltration at 2600 Pa for 20 min, the samples were incubated overnight at 37 °C. Anther samples were then immersed in a fixative solution of 4% sucrose, 1× PBS, 4% paraformaldehyde and 0.25% glutaraldehyde, at 4 °C overnight. The samples were then dehydrated in ethanol series of increasing concentrations (30, 50, 70, 85, 90, 95 and 100%). Tissues were embedded in Technovit® resin, then sectioned on a microtome to a thickness of 8–14 µm, counterstained with ruthenium red and DPX mounted (Sigma, St. Louis, MO) on glass slides. The sections were observed using a LEICA ASLMD laser dissection microscope coupled with a CCD camera (The University of Adelaide microscopy).

### Electron and light microscopy

Sterile (*ms1d*) and fertile (*Ms1*) mature anthers before dehiscence were fixed with either 4% paraformaldehyde, 1.25% glutaraldehyde and 4% sucrose in phosphate-buffered saline (PBS) at pH 7.4, for 16 h at 4 °C for scanning electron microscopy (SEM) or 3% glutaraldehyde in 0.1 M phosphate buffer at pH 7.0 overnight for transmission electron (TEM) or light microscopy. The samples for SEM were rinsed twice with PBS at pH 7.4 for 5 min, whereas the samples for TEM and light microscopy were washed twice with 1 x PBS and embedded in 2% low melting point agarose (Sigma, St. Louis, MO) in 1 x PBS for sample orientation and sectioning, and then dehydrated using a series of graded ethanol solutions (30%, 50%, 70%, 85%, 90% and 95%) each for 60 min. The samples were then infiltrated 3 times, each for 60 min, in 100% ethanol. The samples were either embedded in LR white resin, sectioned (2 µm) and stained with 0.05% toluidine blue stain and mounted on slides in DPX solution (Sigma, St. Louis, MO) for light microscopy or dissected, then they were critical point dried and sputter coated with platinum (BalTec CPD030 Critical Point Dryer) for SEM. 70–80 nm ultrathin anther sections were prepared and stained in 4% uranyl acetate followed by Reynold’s lead citrate (The University of Adelaide microscopy)^43^. SEM and image capture was performed at an accelerating voltage of 10 kV (Philips XL20 SEM w EDAX EDS), whereas TEM and image capture was performed on a Phillips CM-1000 TEM (The University of Adelaide microscopy). Light microscopy images were captured using a Zeiss Axio Imager M2 optical microscope (Zeiss, Germany).

### Fatty acid profiling

Approximately 50 frozen anthers were transferred into pre-chilled cryogenic mill tubes and weighed accurately. A 300 µl aliquot of 1:3:1 chloroform:methanol:water containing a 30 µM internal standard (^13^C_1_ myristic acid) was added to each sample tube. Dried samples and a fatty acid calibration mix (Supelco^®^37 Component FAME Mix) was prepared by adding 25 µl of 2:1 chloroform:methanol followed by shaking at 37 °C for 30 min. The samples were then derivatised using 5 µl of Meth-Prep™ II (Grace Davison Discovery). 1 µl was injected onto the GC column. The GC-MS apparatus comprised of a Gerstel 2.5.2 autosampler, a 7890A Agilent gas chromatograph and a 5975C Agilent quadrupole mass spectrometer (Agilent, Santa Clara, USA). The mass spectrometer was calibrated according to the manufacturer’s recommendations using *tris*-(perfluorobutyl)-amine (CF43).

Gas chromatography was performed on a VF-5ms column (Agilent Technologies, Australia). The injection temperature was set at 250 °C, with the MS transfer line at 280 °C, the ion source adjusted to 250 °C and the quadrupole at 150 °C. Helium was used as the carrier gas at a flow rate of 1.1 ml min^−1^. The corresponding GC-MS method was performed using the following temperature programme; start at an injection of 50 °C, hold for 1 min, followed by a 15 °C min^−1^ oven temperature ramp to 230 °C; hold for 3 min, followed by a 10 °C ramp to 300 °C.

Mass spectra were recorded at 2 scans s^−1^ with an *m/z* value of 50–600 scanning range. Both chromatograms and mass spectra were evaluated using the MassHunter Workstation software version B.07.00 (Agilent, Santa Clara, USA). The retention times and mass spectra (unique qualifier ions) were identified and compared directly to standards from a commercially available fatty acid methyl ester mix (Supelco^®^37 Component FAME Mix, 47885-U, Sigma-Aldrich). All fatty acid methyl esters identified were quantified using prepared calibration curves from the stock Supelco^®^37 Component FAME Mix in the linear range from 2.5 to 150 μM for each lipid class.

### Mapping *Ms1*

Using sequence collinearity among chromosomes 1, 3 and 4 from rice, *Brachypodium* and barley^44^, respectively, we were able to identify gene sequences from these species and use them in a BLASTn search of ESTs and homeogroup four-derived genomic survey sequences from the bread wheat cv. Chinese Spring^45^. Chromosome arm 4AL, 4BS and 4DS-derived genomic sequence contigs were then used to develop PCR-based markers that were subsequently validated for sub-genome specificity by amplification from the Chinese Spring-derived nullisomic tetrasomic set (N4AT4D, N4AT4B, N4BT4A, N4BT4D, N4DT4A and N4DT4B). The region spanning *Ms1* was then identified by amplification of these markers on the sterile deletion mutant series *ms1a*, *ms1b* and *ms1c* ^15–19^ relative to their respective fertile wild-type (*Ms1*) controls^15–19^.

An F_2_-mapping population derived from a cross between the male sterile cv. *Chris*-EMS-mutagenised line FS2 (*ms1d*)^21^ and male fertile cv. *Gladius* ^46^ was developed. *Ms1*-flanking markers identified by deletion mutant mapping were converted into high-resolution melting (HRM) markers^47^ and tested for polymorphism between parental genotypes, and then assayed for segregation within the F_2_ population. Recombinants within the *Ms1* region were identified from F_2_- and F_3_-derived individuals using a combination of both HRM markers and KBioscience competitive allele-specific polymerase chain reaction (KASPar) assays^48^ based on SNPs used to develop both the 9K^49^ and 90K^50^ iSelect Beadchip Assay from Illumina. Primers used for either HRM or KASPar assays are listed in Supplementary Table 5 and 6. All recombinants for the region were marker selected, then fertility tested and not male sterile subsequently progeny tested for fertility in order to determine the zygosity.

### BAC clone analysis

Eighteen probes were designed within the 0.5-cM region bounded by markers wsnp_Ex_c18318_27140346 (*x*27140346) and wsnp_Ku_c7153_12360198 (x12360198), using synteny to *Brachypodium* and rice. The probes were designed to be non-repetitive based on BLAST analysis of target sequences. The probes were then PCR amplified and separated by agarose gel electrophoresis with fragments of desired size being eluted from the gel using a Qiaquick Gel Extraction kit (Qiagen, Germantown, MD, USA). PCR fragments were pooled to an equimolar concentration and then ^32^P-dATP radiolabelled by a NEBlot kit (New England Biolabs) using a manufacturer’s protocol. The labelled probe was purified in a Sephadex G50 column (GE Healthcare) and denatured at 100 °C for 10 min. Twenty eight high-density BAC clone colony filters gridded onto Hybond N + nylon membranes (GE Healthcare, Piscataway, NJ, USA) were used for hybridisation. This represents a coverage of 5.1-genome equivalents from the *Triticum turgidum ssp*. durum wheat cv. Langdon^42^. For prehybridisation, overnight incubation of colony filters in a hybridisation solution (2x SSPE, 0.5% SDS, 5x Denhardt’s reagent^51^ and 40 μg ml^−1^ salmon sperm DNA) was done in rotary glass tubes at 65 °C. The labelled probe was mixed with 5 ml of hybridisation solution and colony filters were incubated at 65 °C overnight. To remove the unbound probe, the filters were washed twice in a washing solution containing 2x SSPE and 0.5% SDS and rinsed with 1x SSC. The washed filters were exposed to an X-ray film for 1–3days based on the signal intensity to identify positive clones. BAC clones that gave a positive signal were grown on single colonies from glycerol stabs and then DNA was extracted according to the BACMAX96^TM^ DNA purification kit (Epicentre®, www.epicentre.com, Madison, Wisconsin, USA).

Restriction mapping, PCR experiments with primers corresponding to the markers previously used, determined the order of the BACs covering the region of interest. BAC libraries from the *Triticum turgidum ssp*. durum cv. Langdon and a bread wheat proprietary cultivar were screened with probes from the *Ms1* region, with positive clones being selected for sequencing. All BACs were sequenced using the Illumina MiSeq platform with paired-end (PE) reads of 250 bp. Quality-controlled PE reads were mapped in a single-end mode to the bread wheat cv. Chinese Spring chromosome arm survey sequencing using Biokanga 2.76.2 (https://github.com/csiro-crop-informatics/biokanga) allowing 2 mismatches per 100 bp to confirm that they were derived from homeogroup 4. The reads were then filtered for bacterial sequence contamination, and trimmed for a vector sequence using a combination of BLASTn-based filters and custom scripts. Before assembly, the overlapping PE reads were fused using FLASH 1.2.7 (https://sourceforge.net/projects/flashpage). The fused reads along with the remaining PE reads were then assembled into contigs using MIRA 4.0^52^. Contigs produced by MIRA were then scaffolded using SSPACE^53^ using mate-pair anchors for each contig derived from a single mate-pair library for all samples. Single contiguous scaffolds for each homeolocus were manually finished using Gap5^54^. Highly repetitive regions on the BACs were masked based on a per-base depth of mapped reads (cv. *Excalibur*, cv. *Gladius* ^55^) exceeding 1000. The alignments to the BACs of *Brachypodium* genes as well as mappings of publically available RNA-seq datasets from the bread wheat cv. Chinese Spring^56^ facilitated gene prediction. Nucleotide sequences spanning the *Ms1* region were submitted to GenBank (accession codes KX447407, KX447408 and KX447409).

### Nucleic acid extraction and expression analysis

DNA extractions from all bread wheat lines were performed using either a phenol/chloroform or freeze-dried extraction protocol^57^. A 15 cm leaf piece from a 2-week-old plant was frozen in liquid nitrogen, and the tissue was ground to a fine powder using one large (9 mm) and three small (3 mm) ball bearings and a vortex. 700 μl of the extraction buffer (1% sarkosyl, 100 mM Tris-HCl at pH 8.5, 100 mM NaCl, 10 mM EDTA and 2% PVPP) was added to each sample and the samples were mixed for 20 min on a rotary shaker. 700 μl of phenol/chloroform/iso-amylalcohol (25:24:1) was added and the extract was transferred to a silica matrix tube and spun at 4000 rpm for 10 min. DNA was precipitated by adding 60 μl of 3 M sodium acetate at pH 4.8 and 600 μl of isopropanol and centrifuged at 13 000 rpm for 10 min. The DNA pellet was washed with 1 ml of 70% ethanol, centrifuged for 2 min at 13 000 rpm and air dried for 20 min. The purified DNA was resuspended in 50 μl of R40 (1x TE, 40 μg ml^−1^ RNase A).

*TaMs1* and homeologous transcripts were detected by qRT-PCR on cDNA using total RNA extracted from wheat of cv. *Chris* (wild type) using an ISOLATE II *RNA* Mini Kit (Bioline). For the anther developmental series, a single anther per floret was squashed in acetocarmine and mounted for microscopy. Microspores were cytologically examined for the stage of development. The remaining two anthers from the same floret were isolated and snap frozen in liquid nitrogen. Developmentally equivalent anthers were pooled and RNA isolated. All total RNA samples were treated with DNase I (Qiagen). First-strand cDNA was synthesised using oligo dT^51^ and Superscript III reverse transcriptase (Thermo Fisher). Amplification products from qRT-PCR on each tissue sample, three technical replicates and three biological replicates were used to estimate *TaMs1* and the homeologue transcript abundance relative to TaEFA2*2, TaGAPdH 2*2 and TaCyclophilin 2*2 reference transcripts. Standard qRT-PCR assays^58^ were performed using primers, as listed in Supplementary Table 7.

### RNA-seq expression analysis

RNA-seq reads derived from five organs (root, leaf, stem, spike and grain) at three developmental stages each from hexaploid wheat cv. Chinese Spring have been published previously^56^. The reads were aligned against the repeat-masked BAC assemblies with Bowtie2^59^ and Tophat2^60^. The returned alignments were stringently filtered so as to remove ambiguously mapped reads and read pairs with conflicting alignments. Gene expression was computed on RNA-seq data by using Cufflinks and Cuffmerge v.3.0^61^. RNA-seq expression data for all predicted coding regions from the BAC assemblies are presented in Supplementary Table 1.

### *TaMs1* sequence from mutant alleles

To identify the *TaMs1* sequence variants in the *ms1d, ms1e* and *ms1f* alleles, the *TaMs1* coding region was amplified from 13 individuals segregating for the sterility phenotype for each mutant allele. PCR used Phusion High-Fidelity DNA Polymerase (NEB, M0530) with Phusion GC buffer, 5% DMSO and 1 M betaine using the primer *TaMs1* (coding region) listed in Supplementary Table 7. The fragments were subcloned into a pCR™8/GW/TOPO® TA Cloning Kit (Thermo Fisher Scientific, K250020) and Sanger sequencing of positive clones was performed by the Australian Genome Research Facility. Sequence chromatograms were compared using Geneious version 6.1.8^62^. Sequence analysis correlated a G-to-A transition at position 329 with the sterility phenotype in the *ms1d* mutant. A Kompetitive Allele-Specific PCR (KASP™) (LGC Genomics) was designed to this SNP transition using Primer Picker (LGC Genomics) (007-0091.1, Supplementary Table 6) and it was assayed using KASP Mastermix on the SNPline (LGC Genomics) on DNA from the *ms1d* x *Gladius* F_2_-mapping population, the *ms1* mutant alleles and across a panel of 192 spring wheat germplasm.

### Phylogenetic analysis

*TaMs1* homologous Poaceae sequences were retrieved from Phytozome (www.phytozome.net), TGAC *Triticum monococcum* Shotgun sequence, International Barley Genome Sequencing Consortium and Rice Genome Annotation Project (http://rice.plantbiology.msu.edu). All BLASTn, BLASTp, tBLASTn and BLASTx hits were retrieved using a cutoff e-value of ≤ 1 × 10^−5^. Default BLAST settings were used for querying with complete sequences. Two prediction tools, PredGPI (gpcr.biocomp.unibo.it/predgpi/) and big-PI Plant Predictor (mendel.imp.ac.at/gpi/plant_server.html), were then used to determine whether primary peptide sequences contained a putative GPI-anchored motif at the C-termini. Protein multiple-sequence alignments (MSAs) were generated using MUSCLE (default settings) implemented in Geneious analysis package (www.geneious.com). Manual alignment was performed to improve the MSAs. The phylogenetic tree was computed with MEGA7 using the maximum likelihood method under default parameters (www.megasoftware.net).

### Constructs

A 4.3-Kb genomic fragment containing approximately 1.5 Kb upstream of *TaMs1* start codon and 1 Kb downstream of the stop codon was synthesised and introduced upstream of the visible marker MoPAT-DsRED (a translational fusion of the bialaphos resistance gene, phosphinothricin-N-acetyl-transferase, and the red fluorescent protein DsRED) transcribed by the maize Ubiquitin promoter^63^ and this 8.1 kb DNA fragment replaced the 2.1 kb *Hind III*-*Eco RI* DNA fragment from PHP43534^64^. This plasmid was introduced into *A. tumefaciens* strain LBA4404^65^ by electroporation using a Gene Pulser II (Bio-Rad)^66^ and used for complementing the *ms1d* mutant. A transcriptional reporter construct containing 1.5 Kb of *TaMs1* promoter sequence was fused to *gusplus* ^67^ and subcloned into the binary vector pMBC32.

### Transformation and in vitro culture

Male-sterile transformation-amenable spring wheat lines, which contain the *ms1d* allele, were developed by backcrossing with pollen from the spring wheat-transformable cv. *Gladius*. Either immature embryos that segregated for the *ms1d* allele (3:1) or the cv. *Fielder* were used as donor materials for transformation. cv. *Fielder* donor material was used to test the transcriptional fusion construct with the *gusplus* reporter gene. Here, *A. tumefaciens* strain ALG0 (pBGXI) was engineered to contain a disarmed pTiBo542 carrying the *TaMs1*::*gusplus* cassette in a pMBC32 backbone. Segregating *ms1d* donor material enabled the transformation directly into a homozygous *ms1d* background to test for genetic complementation of the *ms1d* mutation with the putative *Ms1* allele in the transformed plants (T_0_). To generate wheat transformants^68^ for testing either complementation of the *ms1* mutation or the *TaMs1* transcriptional reporter, wheat (*Triticum aestivum* L., cv. *Gladius* or cv. *Fielder*) plants were grown in a growth chamber at 18/15 °C (day/night), with a 16-hr photoperiod (minimum of 1000 μmol s^−1^ m^−2^ light) or in a greenhouse. Immature seeds with immature embryos (IEs) of about 1.5–2.5 mm collected from spikes 12–14 days post anthesis were surface-sterilised for 20 min in 15% (v v^−1^) bleach (5.25% sodium hypochlorite) plus one drop of Tween 20 followed by three washes in sterile water. IEs were isolated and placed in 1.0 ml of liquid infection medium (WI4; MS salt + vitamins (4.43 g l^−1^), maltose (30 g l^−1^), glucose (10 g l^−1^), MES (1.95 g l^−1^), 2,4-D (1 ml, 0.5 mg l^−1^), Picloram (200 µl, 10 mg ml^−1^) and BAP (0.5 ml, 1 mg l^−1^)) with 0.25 ml of autoclaved sand into 2 ml microcentrifuge tubes. IEs were treated by centrifuging at various strengths in an infection medium and then inoculated with *Agrobacterium*. The suspension of *Agrobacterium* and IEs was poured onto a co-cultivation medium (a WI4 medium containing 5.0 µM CuSO_4_ without glucose solidified with 3.5 g l^−1^ of Phytagel). IEs were then placed on an embryo axis side down on the media, and incubated in the dark at 21 °C. After three days, IEs were transferred to a DBC4 medium containing 100 mg l^−1^ of cefotaxime (PhytoTechnology Lab., Shawnee Mission, KS) and then incubated at 26–28 °C under dim light for two weeks. The DBC4 medium is a DBC3 green-regenerative medium^69^ modified with 1.0 mg l^−1^ of 6-benzylaminopurine (BAP). The tissues were then transferred to a DBC6 medium (a modified DBC3 medium with 0.5 mg l^−1^ of 2,4-dichlorophenoxyacetic acid and 2.0 mg l^−1^ of BAP) containing 150 mg l^−1^ of cefotaxime for another two weeks. Regenerable *DsRed-* expressing transgenic sectors were identified using a Leica M165 FC fluorescence microscope, cut from the non-transformed tissues and placed on a MSA regeneration medium [MSB^68^ without indole-3-butyric acid] with 150 mg l^−1^ of cefotaxime, whereas *TaMs1::gusplus*-containing tissues were selected based on resistance to 100 mg l^−1^ of hygromycin B. After sectors have developed into small plantlets, they were transferred to an MSB-rooting medium. During each transfer, plantlets were checked for *DsRed* gene expression and any non-expressing or chimeric tissues were removed.

### T_0_ plantlet generation and analysis

T_0_ wheat regenerants containing a single- or multicopy *TaMs1*–*DsRed* cassette(s) were identified by copy number qPCR^70^ using the following forward, reverse and probe primers (Fwd: 5′-GACATCCCCGACTACAAGAAGCT-3′, Rev: 5′-CACGCGCTCCCACTTGA-3′ and Probe1-FAM MGB 5′-CCTTCCCCGAGGGC-3). Zygosity was shown of the *ms1d* mutation using flanking and linked markers (Supplementary Table 5 and 6). Plantlets were transferred to the glasshouse and assessed for self-fertility and expression of *DsRed* fluorescence in the resulting seed. Seed counts from these individual plants were counted as a qualitative measure of male fertility.

### Molecular and phenotypic traits of T_1_ plants

Inheritance of complementation by *TaMs1* T-DNA insertion was shown by analysing a selfed seed set on T_1_ plants derived from two separate T_0_ plants, each with independent T-DNA insertions (Event-1 and Event-7) (Supplementary Table 4). One set of T_1_ progenies was derived from a T_0_ plant homozygous for *ms1d* mutation (*ms1d*/*ms1d*) and containing the *TaMs1*–*DsRed* cassette (Event-1). The second set of T_1_ progenies was derived from a T_0_ plant heterozygous for *ms1d* mutation (*Ms1*/*ms1d*) and containing the *TaMs1*–*DsRed* cassette (Event-7). Genotyping for *ms1d* zygosity and the presence of the T-DNA insertion for plants derived from both sets were determined using flanking markers, as described above, and the expression of the *DsRed* seed colour marker.

### Data availability

MiSeq BAC-sequencing data have been deposited in the NCBI SRA database under Bioproject ID PRJNA396428. The assembled genomic DNA of BACs derived from the *Ms1* locus (KX447407) as well as its A (KX447408) and D (KX447409) genome-derived homeoloci have been deposited in NCBI GenBank. Further data that support the findings of this study are available from the corresponding author upon reasonable request.

## Electronic supplementary material

Supplementary Information

Peer Review File

## References

[1] FAOSTAT. http://faostat3.fao.org (2013–2015).

[2] HY-WHEAT. Scientific and technological cooperation in plant genome research as basis of the ‘Knowledge-Based Bio-Economy’ (Plant - KBBE) 2010 Project HY-WHEAT, http://www.agence-nationale-recherche.fr/?Project=ANR-10-KBBE-0004 (2010).

[3] Longin CFH (2013). Hybrid wheat: quantitative genetic parameters and consequences for the design of breeding programs. Theor. Appl. Genet..

[4] Muhleisen J, Piepho HP, Maurer HP, Longin CF, Reif JC (2014). Yield stability of hybrids versus lines in wheat, barley, and triticale. Theor. Appl. Genet..

[5] Longin CFH, Reif JC, Würschum T (2014). Long-term perspective of hybrid versus line breeding in wheat based on quantitative genetic theory. Theor. Appl. Genet..

[6] Whitford R (2013). Hybrid breeding in wheat: technologies to improve hybrid wheat seed production. J. Exp. Bot..

[7] Driscoll CJ (1972). XYZ system of producing hybrid wheat. Crop Sci..

[8] Driscoll CJ (1985). Modified XYZ system of producing hybrid wheat. Crop Sci..

[9] Wu Y (2015). Development of a novel recessive genetic male sterility system for hybrid seed production in maize and other cross-pollinating crops. Plant Biotechnol. J..

[10] Druka A (2011). Genetic dissection of barley morphology and development. Plant Physiol..

[11] Franckowiak J (1997). Revised linkage maps for morphological markers in barley, *Hordeum vulgare*. Barley Genet. Newslett.

[12] Cigan AM (2016). Targeted Mutagenesis of a conserved anther‐expressed P450 gene confers male sterility in monocots. Plant Biotechnol. J..

[13] Klindworth DL, Williams ND, Maan SS (2002). Chromosomal location of genetic male sterility genes in four mutants of hexaploid wheat. Crop Sci..

[14] Pugsley AT, Oram RN (1959). Genetic male sterility in wheat. Aust. Plant Breed. Genet. Newslett..

[15] Driscoll CJ (1975). Cytogenetic analysis of two chromosomal male-sterility mutants in hexaploid wheat. Aust. J. Biol. Sci..

[16] Fossati A, Ingold M (1970). A male sterile mutant in Triticum aestivum. Wheat Inform. Serv..

[17] Barlow KK, Driscoll CJ (1981). Linkage studies involving two chromosomal male-sterility mutants in hexaploid wheat. Genetics.

[18] Driscoll CJ (1977). Registration of cornerstone male sterile wheat germplasm (reg. no. GP 74). Crop Sci..

[19] Kleijer G, Fossati A (1976). Chromosomal location of a gene for male sterility in wheat (*Triticum aestivum*). Wheat Inform. Serv..

[20] Endo TR, Mukai Y, Yamamoto M, Gill BS (1991). Physical mapping of a male-fertility gene of common wheat. Jpn. J. Genet..

[21] Sasakuma T, Maan SS, Williams ND (1978). EMS-induced male-sterile mutants in euplasmic and alloplasmic common wheat. Crop Sci..

[22] Briggle L (1970). A recessive gene for male sterility in hexaploid wheat. Crop Sci..

[23] Islam A, Driscoll C (1984). Latent male fertility in’Cornerstone’chromosome 4A. Can. J. Genet. Cytol..

[24] Joshi GP, Li J, Nasuda S, Endo TR (2013). Development of a self-fertile ditelosomic line for the long arm of chromosome 4B and its characterization using SSR markers. Genes Genet. Syst..

[25] Borner GHH, Lilley KS, Stevens TJ, Dupree P (2003). Identification of glycosylphosphatidylinositol-anchored proteins in *Arabidopsis*. A proteomic and genomic analysis. Plant Physiol..

[26] Kim H (2012). Characterization of glycosylphosphatidylinositol-anchored lipid transfer protein 2 (*LTPG2*) and overlapping function between *LTPG*/*LTPG1* and *LTPG2* in cuticular wax export or accumulation in *Arabidopsis thaliana*. Plant Cell Physiol..

[27] Franckowiak JD, Maan SS, Williams ND (1976). A proposal for hybrid wheat utilizing *Aegilops squarrosa* L. cytoplasm. Crop Sci..

[28] José-Estanyol M, Gomis-Rüth FX, Puigdomènech P (2004). The eight-cysteine motif, a versatile structure in plant proteins. Plant Physiol. Biochem..

[29] Dong C, Dalton-Morgan J, Vincent K, Sharp P (2009). A modified TILLING method for wheat breeding. Plant Genome.

[30] Zhou K, Wang S, Feng Y, Liu Z, Wang G (2006). The 4E-system of producing hybrid wheat. Crop Sci..

[31] Wang S, Zhou K, Yang W, Wang H (2013). Technical difficulties and solutions of applying 4E-*ms* system in hybrid wheat production. J. Triticeae Crops.

[32] Zhang, D., Shi, J. & Yang, X. in *Lipids in Plant and Algae Development*, 315–337 (Springer, 2016)

[33] Yang, L. et al. *Induced Plant Mutations in the Genomics Era*. 370–372 (Food and Agriculture Organization of the United Nations, 2009).

[34] Cao W, Somers DJ, Fedak G (2009). A molecular marker closely linked to the region of *Rht-D1c* and *Ms2* genes in common wheat (*Triticum aestivum*). Genome.

[35] Maan S, Kianian S (2001). Third dominant male sterility gene in common wheat. Serv.

[36] Ni F (2017). Wheat *Ms2* encodes for an orphan protein that confers male sterility in grass species. Nat. Commun..

[37] Xia C (2017). A TRIM insertion in the promoter of *Ms2* causes male sterility in wheat. Nat. Commun..

[38] Singh M, Kumar M, Thilges K, Cho M-J, Cigan AM (2017). MS26/CYP704B is required for anther and pollen wall development in bread wheat (*Triticum aestivum* L.) and combining mutations in all three homeologs causes male sterility. PLoS ONE.

[39] Voytas DF, Gao C (2014). Precision genome engineering and agriculture: opportunities and regulatory challenges. PLoS Biol..

[40] Svitashev S (2015). Targeted mutagenesis, precise gene editing, and site-specific gene insertion in maize using Cas9 and guide RNA. Plant Physiol..

[41] Sears, E. R. in *Chromosome Manipulations and Plant Genetics*, 29–45 (Springer, 1966).

[42] Cenci A (2003). Construction and characterization of a half million clone BAC library of durum wheat (*Triticum turgidum* ssp. durum). Theor. Appl. Genet..

[43] Reynolds ES (1963). The use of lead citrate at high pH as an electron opaque stain in electron microscopy. J. Cell. Biol..

[44] Mayer KF (2011). Unlocking the barley genome by chromosomal and comparative genomics. Plant Cell.

[45] Mayer KF (2014). A chromosome-based draft sequence of the hexaploid bread wheat (Triticum aestivum) genome. Science.

[46] Australian Grain (2007). Technologies Pty. Ltd. Gladius. Plant Varieties J..

[47] Tucker EJ, Huynh BL (2014). Genotyping by high-resolution melting analysis. Crop Breed. Methods Protoc..

[48] He, C., Holme, J. & Anthony, J. in *Methods in Molecular Biology*, Vol. 1145 (eds D. Fleury & R. Whitford) Ch. 7, 75-86 (Springer, 2014).

[49] Cavanagh CR (2013). Genome-wide comparative diversity uncovers multiple targets of selection for improvement in hexaploid wheat landraces and cultivars. Proc. Natl Acad. Sci..

[50] Wang S (2014). Characterization of polyploid wheat genomic diversity using a high‐density 90 000 single nucleotide polymorphism array. Plant. Biotechnol. J..

[51] Green, M. R. & Sambrook, J. *Molecular Cloning: a Laboratory Manual*, Vol. 1 (Cold Spring Harbor Laboratory Press, 2012).

[52] Chevreux, B., Wetter, T. & Suhai, S. in *German Conference on Bioinformatics*. 45–56. (Hannover, Germany, 1999)

[53] Boetzer M, Henkel CV, Jansen HJ, Butler D, Pirovano W (2011). Scaffolding pre-assembled contigs using SSPACE. Bioinformatics.

[54] Bonfield JK, Whitwham A (2010). Gap5—editing the billion fragment sequence assembly. Bioinformatics.

[55] Edwards D (2012). Bread matters: a national initiative to profile the genetic diversity of Australian wheat. Plant Biotechnol. J..

[56] Choulet F (2014). Structural and functional partitioning of bread wheat chromosome 3B. Science.

[57] Kovalchuk, N. in *Methods in Molecular Biology*, Vol. 1145 (eds D. Fleury & R. Whitford) 239–252 (Springer, 2014).

[58] Burton RA, Shirley NJ, King BJ, Harvey AJ, Fincher GB (2004). The CesA gene family of barley. Quantitative analysis of transcripts reveals two groups of co-expressed genes. Plant Physiol.

[59] Langmead B, Salzberg SL (2012). Fast gapped-read alignment with Bowtie 2. Nat. Methods.

[60] Kim D (2013). TopHat2: accurate alignment of transcriptomes in the presence of insertions, deletions and gene fusions. Genome Biol..

[61] Trapnell C (2012). Differential gene and transcript expression analysis of RNA-seq experiments with TopHat and Cufflinks. Nat. Protoc..

[62] Kearse M (2012). Geneious basic: an integrated and extendable desktop software platform for the organization and analysis of sequence data. Bioinformatics.

[63] Ananiev EV (2009). Artificial chromosome formation in maize (Zea mays L.). Chromosoma.

[64] Rudrappa, T. & Girhepuje, P. V. Method of sunflower regeneration and transformation using radicle free embryonic axis. US patent US8901377 (2014).

[65] Komari T, Hiei Y, Saito Y, Murai N, Kumashiro T (1996). Vectors carrying two separate T-DNAs for co-transformation of higher plants mediated by Agrobacterium tumefaciens and segregation of transformants free from selection markers. Plant J..

[66] Gao H (2010). Heritable targeted mutagenesis in maize using a designed endonuclease. Plant J..

[67] Vickers CE, Schenk PM, Li D, Mullineaux PM, Gresshoff PM (2007). pGFPGUSPlus, a new binary vector for gene expression studies and optimising transformation systems in plants. Biotechnol. Lett..

[68] Ishida Y, Tsunashima M, Hiei Y, Komari T (2015). Wheat (Triticum aestivum L.) transformation using immature embryos. Methods Mol. Biol..

[69] Cho, M. J., Ellis, S. R., Gordon-Kamm, W. J. & Zhao, Z. Y. *Methods and compositions for producing and selecting transgenic plants* Worldwide patent WO2014093485 (2014).

[70] Fletcher SJ (2014). qPCR for quantification of transgene expression and determination of transgene copy number. Crop Breed. Methods Protoc..

