# Supplementary material for: Molecular identification of the wheat male fertility gene Ms1 and its prospects for hybrid breeding
Source: Nat Commun. 2017 Oct 11;8:869. doi: 10.1038/s41467-017-00945-2 (PMC5636796; doi:10.1038/s41467-017-00945-2)
Supplement: Supplementary file 1 — Supplementary Information [file 41467_2017_945_MOESM1_ESM.pdf]

File name: Supplementary Information
Description: Supplementary Figures and Supplementary Tables

File name: Peer Review File

Supplementary Information

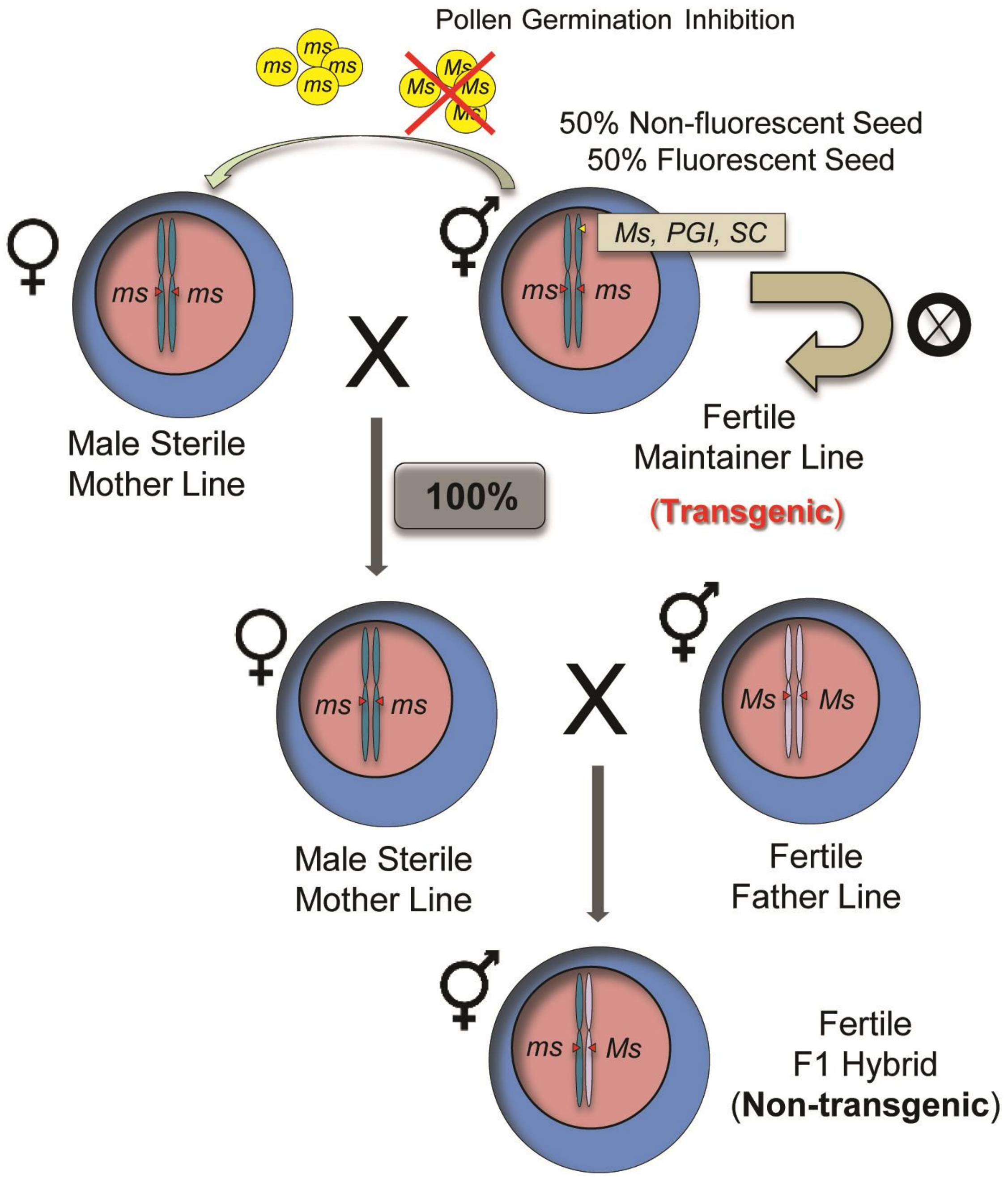

Supplementary Fig. 1: A schematic of the genetic components contributing to the Seed Production Technology hybridization platform which uses a transgenic maintainer line for the propagation of homozygous recessive male sterile mutant mother lines. The maintainer transgene contains a dominant fertility restorer (*Ms*), a seed colour marker (*SC*) and is biologically contained to the maintainer line through the action of a pollen germination inhibitor (*PGI*). *SC* allows the visual separation of transgenic maintainer seed from non-transgenic male sterile seed. F1 hybrids produced from this process are non-transgenic.

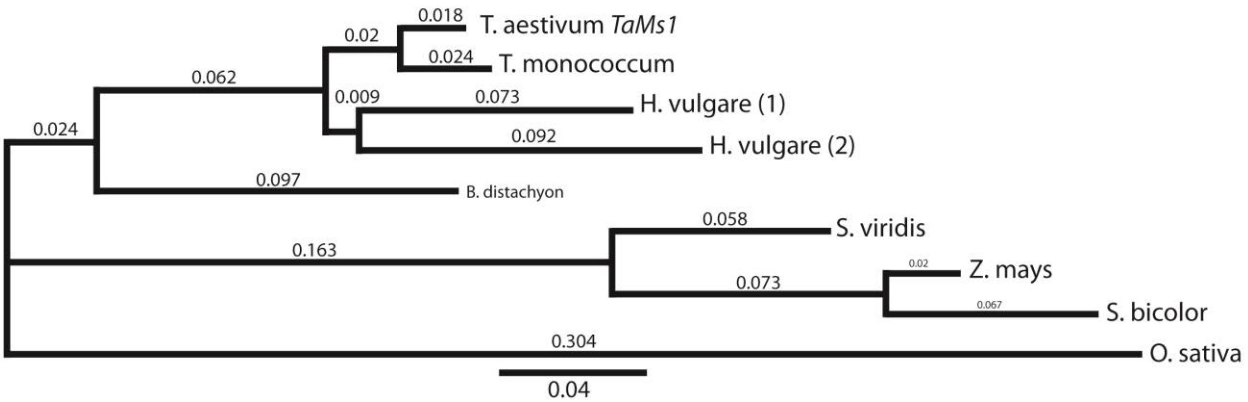

Supplementary Fig. 2: Molecular phylogenetic analysis for *TaMs1* by Maximum Likehood method. Evolutionary history was inferred by using the Maximum Likelihood method based on the JTT matrix-based model. The tree with the highest log likelihood (-1621.4402) is shown. Initial tree(s) for the heuristic search were obtained automatically by applying Neighbor-Join and BioNJ algorithms to a matrix of pairwise distances estimated using a JTT model, and then selecting the topology with superior log likelihood value. The tree is drawn to scale, with branch lengths measured in the number of substitutions per site (next to the branches). The analysis was performed on 7 primary polypeptide sequences derived from *T. aestivum* (*TaMs1*), *T. monococcum* (TGAC v1_contig81546:22-1765), *H. vulgare* (1- chr4H:5322056-53239903, 2- chr4H:5283435-5285262), *B. distachyon* (Bradi1g13030), *S. viridis* (Sevir.7G115900.1), *Z. mays* (GRMZm2g151021), *S. bicolor* (Sb06g017510) and *O. sativa* (LOC_Os03g46110). All positions containing gaps and missing data were eliminated. A total of 195 positions are in the final dataset. Evolutionary analyses were conducted in MEGA7.

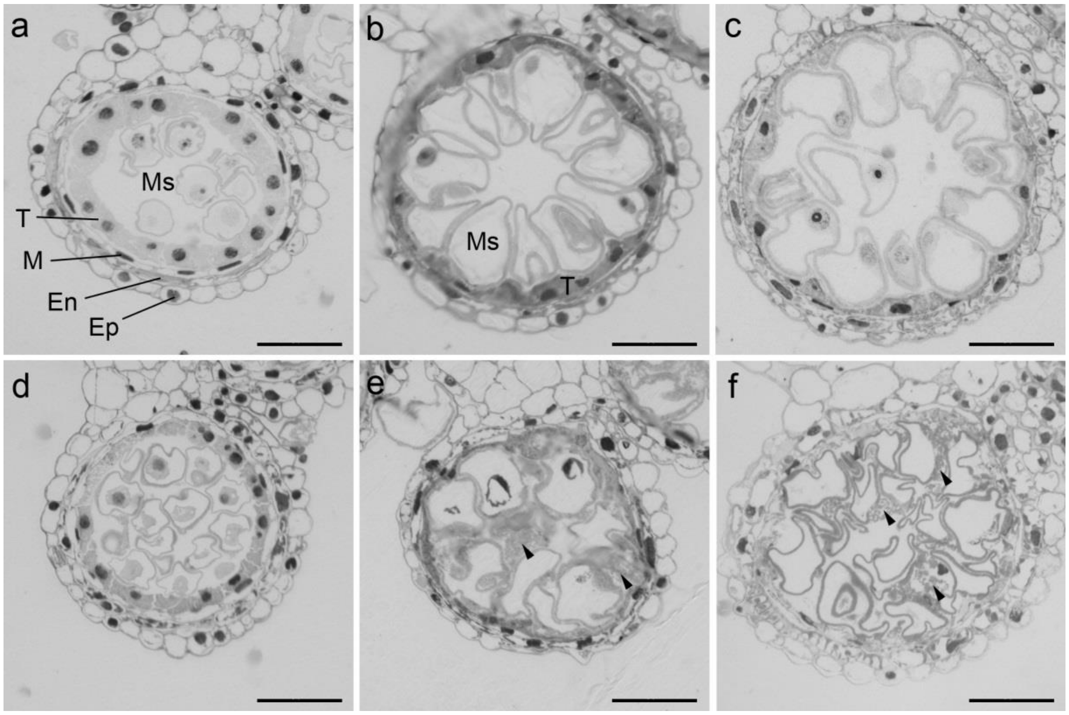

Supplementary Fig. 3: Microspore development in wild-type versus mutant anthers between uninucleate microspore and bicellular pollen stage. Transverse sections of anthers from wild type (*Ms1*, a to c) and *ms1d* (d to f) were examined by compound light microscopy at different developmental stages: (a and d) early uninucleate microspore stage, (b and e) late uninucleate microspore stage, (c and f) early bicellular pollen stage. Aggregated exine components are depicted in *ms1d* anther sections by arrowheads in panels (e) and (f). Abbreviations: En, endothecial cell; Ep, epidermal cell, M, middle layer; Ms, microspore; T, tapetum cell. Scale bars = 50 µm.

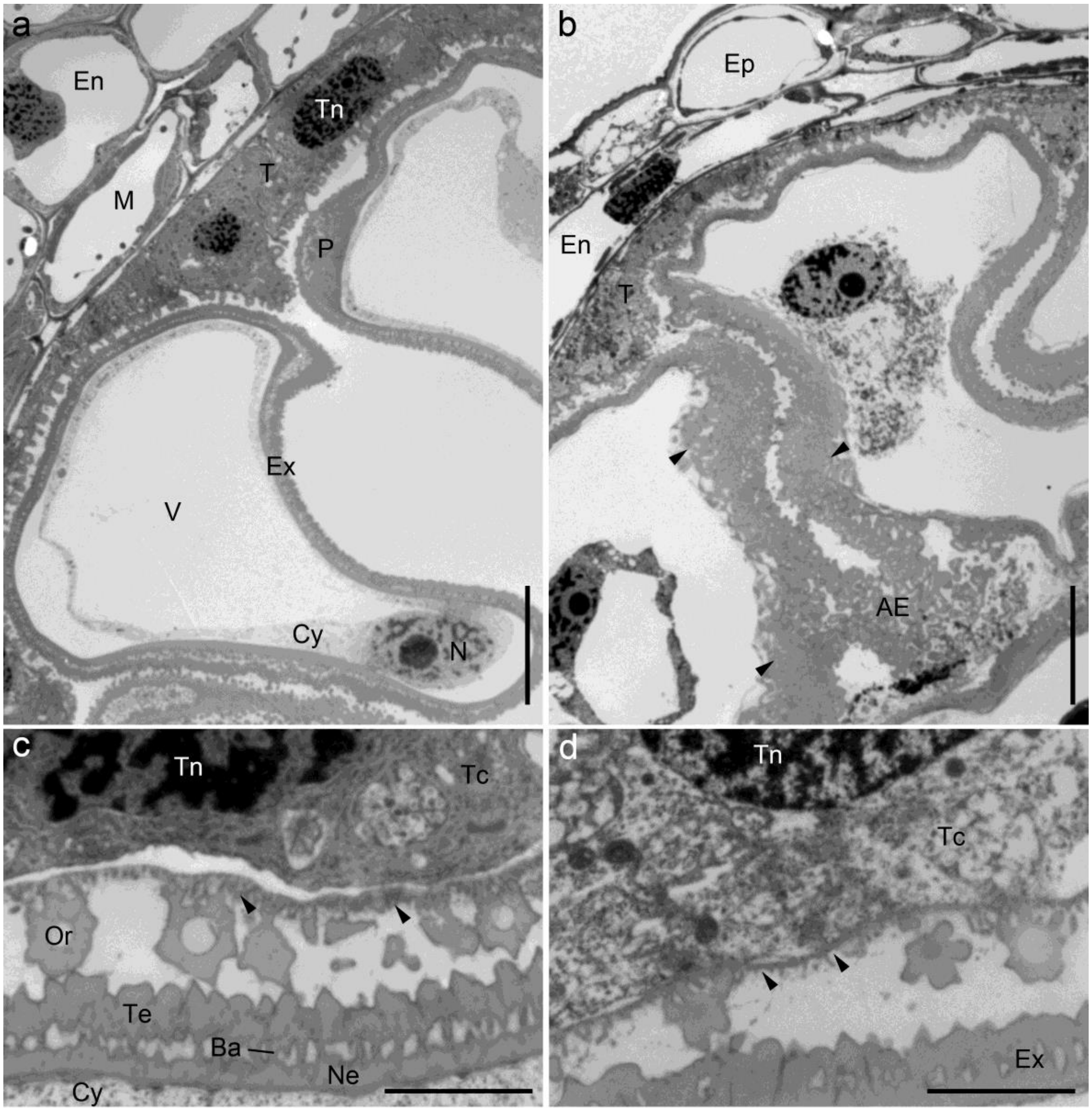

Supplementary Fig. 4: Transmission electron micrographs of microspores and tapetal cells from wild type versus mutant anthers. Representative transmission electron micrographs of wild type (*Ms1*, a and c) and *ms1d* (b and d) anther cross-sections containing microspores at late uninucleate stage (a and b) and early bicellular pollen stage (c and d). Arrowheads in panels indicate ectopic exine deposition (b) and less electron dense material at the tapetal cell surface (d) in *ms1d* relative to wild-type (a c). Abbreviations: AE, aggregated exine components; Ba, bacula; Cy, cytoplasm; En, endothecial cell; Ep, epidermal cell, M, middle layer; Ms, microspore; Or, orbicule; T, tapetum cell; Tc, tapetum cytoplasm; Te, tectum; Tn, tapetum nucleus; V, vacuole. Scale bars in (a) and (b) = 10 µm and in (c) and (d) = 2 µm.

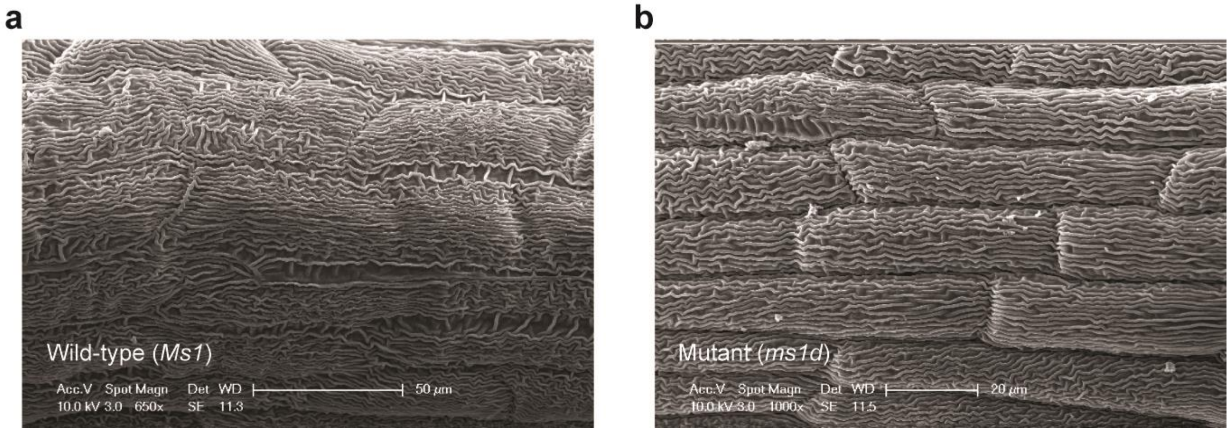

Supplementary Fig. 5: Scanning electron micrographs of the epiderminal cuticle layer from wild-type and mutant anthers. No observable defects in the cutical surface layer of (a) Wild-type male fertile (*Ms1*) versus (b) male sterile (*ms1d*) anthers. Scale bars in (a) = 50 µm and in (b) = 20 µm.

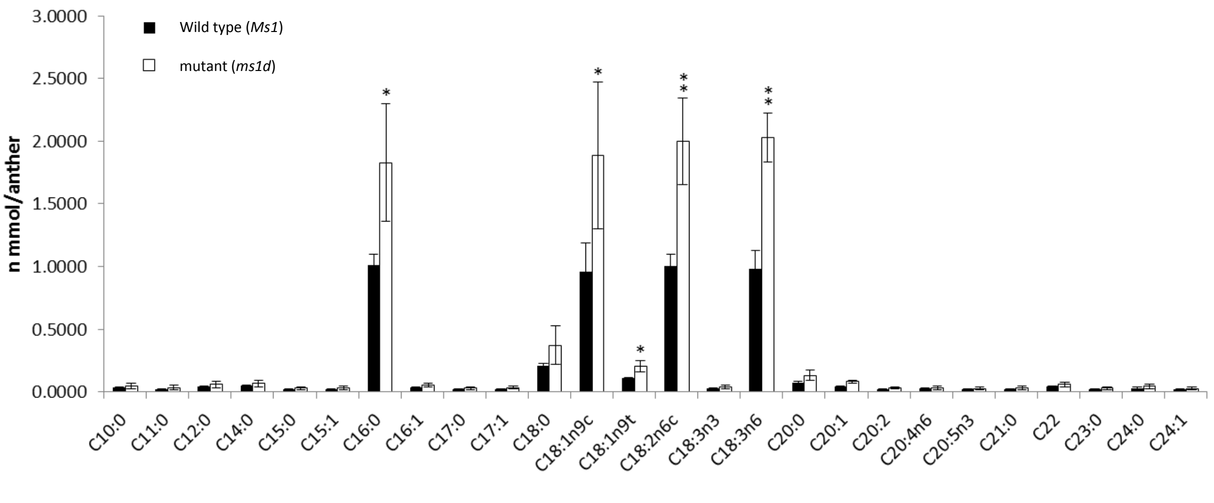

Supplementary Fig. 6: Accumulation of long chain fatty acids in *ms1d* anthers containing pre-meiotic to uninucleate microspores relative to wild-type. Compounds are abbreviated as follow: C10:0, Capric Acid; C11:0, Undecanote Acid ; C12:0, Lauric Acid, C13:0, Tridecanoate; C14:0, Myristic Acid; C14:1, Myristoleic Acid ; C15:0, Pentadecanoic Acid ; C15:1, cis-10-Pentadecanoic acid ; C16:0, Palmitic Acid; C16:1, Palmitoleic Acid; C17:0, Heptadecanoic Acid; C17:1, cis-10-Heptadecenoic acid; C18:0, Stearic Acid; C18:1n9c, Oleic Acid; C18:1n9t, Elaidic Acid, C18:2n6; Linoleic Acid; C18:2n6t, Linolelaidic Acid; C18:3n3, α-Linolenic acid; C18:3n6, γ-Linolenic Acid; C20:0, Arachidic Acid; C20:1, cis-11-Eicosenoic Acid; C20:2, cis-11,14-Eicosadienoic acid; C20:3n6, cis-8,11,14-Eicosatrienoic acid ; C21:0, Heneicosanoic Acid; C22:0, Behenic Acid; C22:1n9, Erucic Acid; C22:6n3, cis-4,7,10,13,16,19-docosahexaenoic Acid; C23:0, Tricosanoic Acid and C24:0, Lignoveric Acid. Error bars indicate SD (n=3). Asterisks (*P<0.05, **P<0.01) denote statistical differences with respect to the wild-type using Student's t-test.

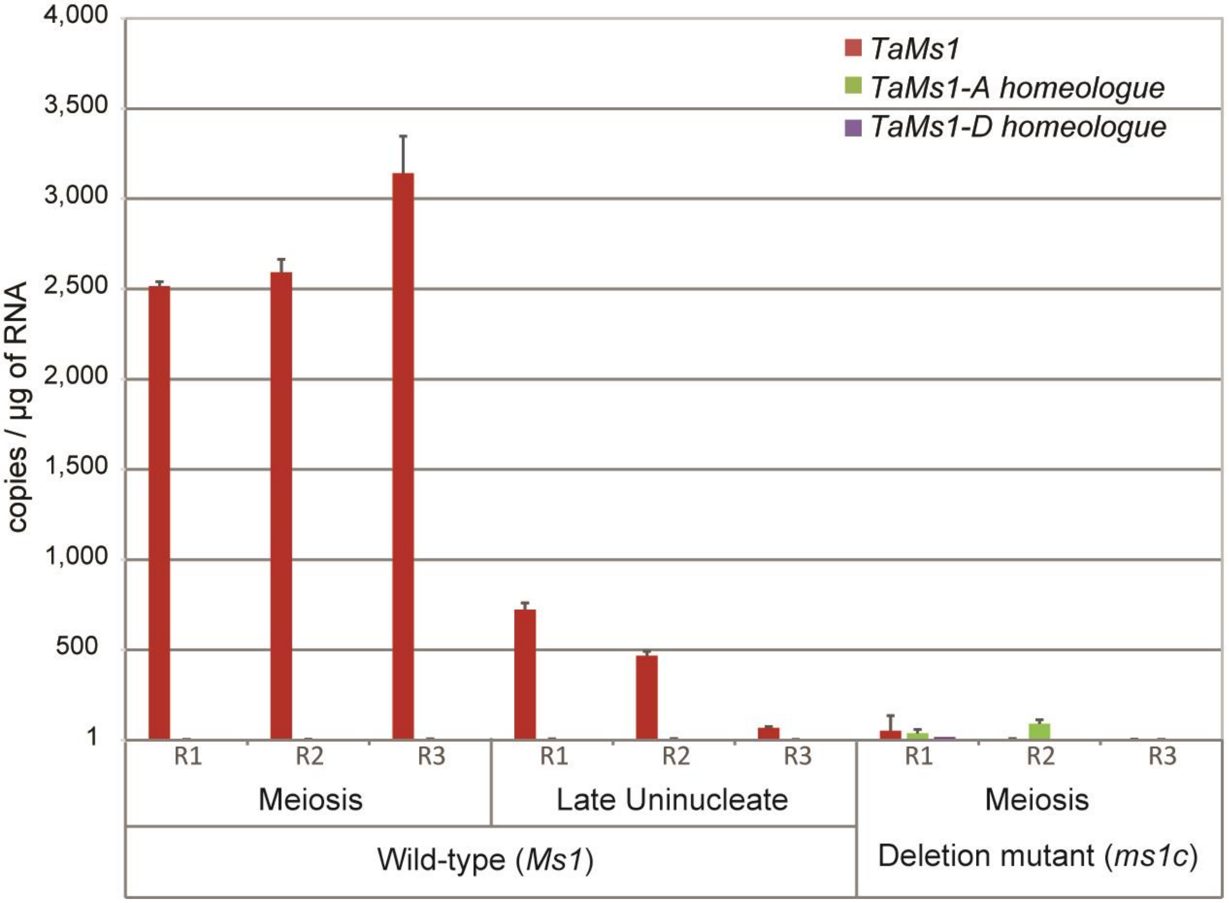

Supplementary Fig. 7: *TaMs1* and homeologue mRNA levels as detected by qRT-PCR in meiotic to late uninucleate microspore containing anthers of cultivar Cornerstone either wild-type for *Ms1* or homozygous for the *ms1c* deletion. R1-R3 represent independent biological replicates. Data are means ± s.e.m (n = 3 biological replicates).

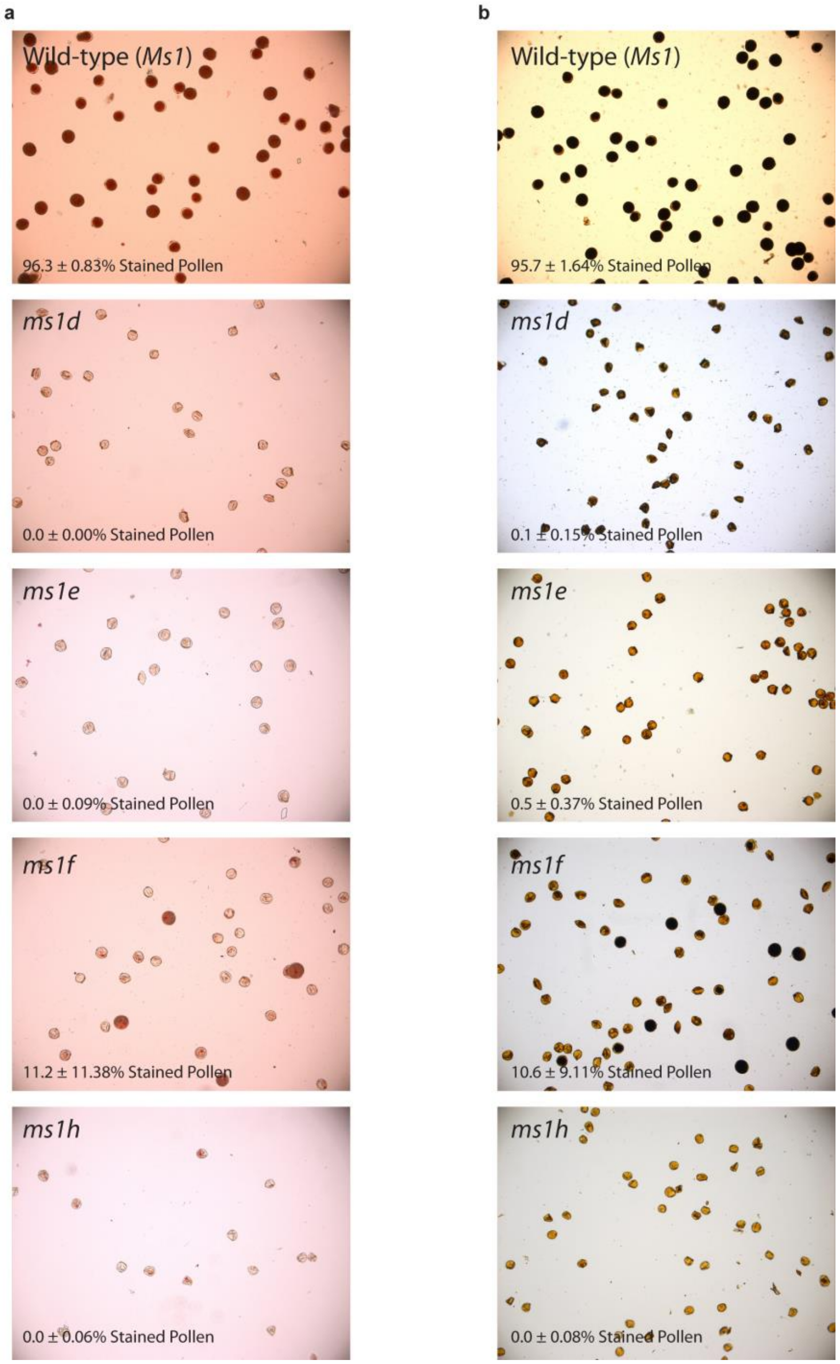

Supplementary Fig. 8: Variation in fertility penetrance between different mutant alleles. Representative brightfield images of mutant (*ms1d*, *ms1e*, *ms1f* and *ms1h*) and wild-type (*Ms1*) pollen grains either (a) acetocarmine-glycerin or (b) Lugol stained for pollen viability. Data presented in the bottom left corner of each panel is the mean percentage ± standard deviation of stained versus non-stained pollen (n = >500 pollen grains counted per sample, 9 biological replicates per genotype). Images were captured at 150x magnification.

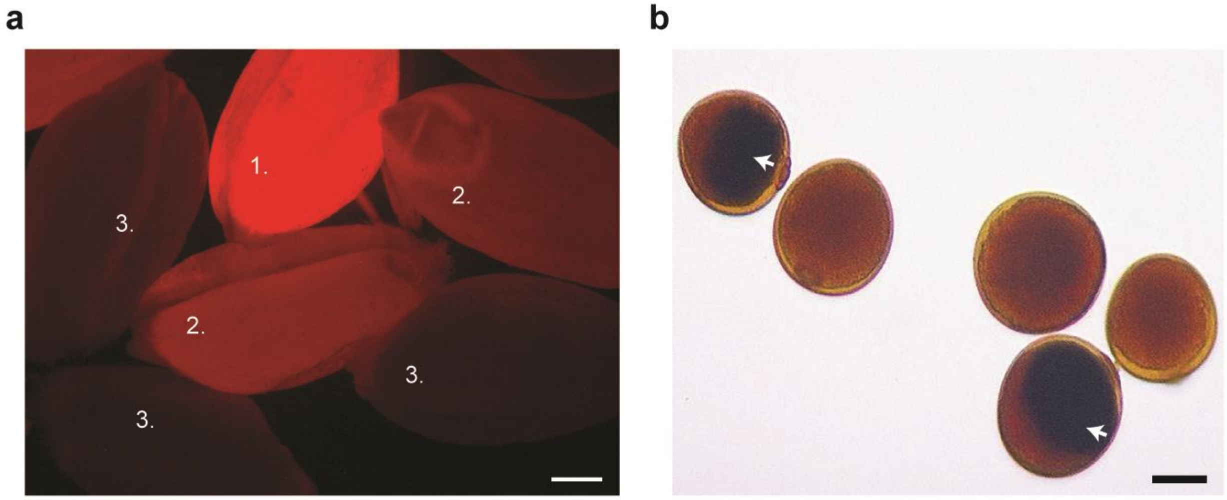

Supplementary Fig. 9: Functionality of both the maize α-amylase gene and *dsRed* seed colour marker in wheat. (a) Wheat seeds expressing a *CaMV35SenhHvLTP2::dsRed* transgene in either the homozygote (1) or heterozygote (2) condition relative to non-transgenic null segregants (3). (b) Wheat pollen grains expressing a *PG47::Zm-α-amylase* transgene lack starch relative to non-transgenic null segregants which appear dark brown/black when stained with Lugol reagent (arrows). Scale bars in (a) = 1 mm and in (b) 50 µm.

Supplementary Table 1: RNA-seq expression data log2 transformed (normalised FPKMs) for predicted coding regions (Genes I-XI) contained within a 304 Kb assembly of sequence derived from the *Ms1* locus of both *Triticum turgidum ssp.* durum and *Triticum aestivum*.

					Grain						Leaf						Root						Spike						Stem					
				Zadock's stage	Z71		Z75		Z85		Z10		Z23		Z71		Z10		Z13		Z39		Z32		Z39		Z65		Z30		Z32		Z65	
Gene	*Brachypodium* Orthologue	Gene Annotation	Pseudoname	Physical Position on BAC assembly	Rep1	Rep2	Rep1	Rep2	Rep1	Rep2	Rep1	Rep2	Rep1	Rep2	Rep1	Rep2	Rep1	Rep2	Rep1	Rep2	Rep1	Rep2	Rep1	Rep2	Rep1	Rep2	Rep1	Rep2	Rep1	Rep2	Rep1	Rep2	Rep1	Rep2
XI	Bradi1g12960	DUF581 domain containing protein	XLOC_000007	43004-46058	0.761	1.207	-0.785	-0.999	-0.700	-1.058	-0.224	-0.620	1.268	1.077	0.382	-0.088	1.139	0.834	3.640	2.895	3.229	2.790	3.503	3.765	3.583	3.631	2.331	2.323	4.760	4.339	3.391	3.408	4.314	4.224
X	Bradi1g12970	Putative GNAT family acetyltransferase	XLOC_000019	46172-46334													1.676		2.163			1.869			1.686								1.820	2.110
IX	Bradi1g12980	Putative parafibromin	XLOC_000009	65752-70804	0.409	-0.019	-1.235	-1.128	0.103	0.409	-0.252	-0.009	-1.038	-1.325	-0.953	-0.787	0.101	-0.103	-0.118	0.111	0.398	-0.212	0.776	0.789	0.153	0.094	0.179	0.128	0.923	-0.095	-0.402	-0.638	-0.737	-0.629
VIII	Bradi1g12990	Lipid Transfer Protein-Like 71	XLOC_000014	68671-70804	-1.524	-1.456	-1.836	-1.845	-1.261	-1.574	-1.923	-1.671	-1.805	-1.569			-1.864	-1.795			-1.821	-1.560	-1.270	-1.253	-1.665	-0.984	-1.283	-1.271	-0.984	-1.349	-0.993	-1.583	-1.155	-1.483
VII	Bradi1g13000	Lipid Transfer Protein-Like 72	XLOC_000015	72388-73451	-0.968	-0.683	-0.721	-0.551			1.451	1.052	0.372	0.398			2.834	2.523	2.698	2.192	2.070	1.382			0.278	-0.349	-0.511	-0.750	0.240	-0.058			-0.039	-0.475
VI	Bradi1g69240	U-box domain containing protein (likely pseudogene)			No reads																													
V	Bradi4g44760	F-box/LRR-repeat protein 3	XLOC_000016	74703-76197	0.582	0.103	0.221	0.112	0.914	0.309	0.182	-0.168	0.175	0.035	0.137	-0.257	0.516	0.597	0.745	0.372	-0.050	0.046	0.557	1.027	0.565	0.829	0.662	0.564	0.614	0.490	0.480	0.195	0.906	0.664
IV (*TaMs1*)	Bradi1g13030	Lipid Transfer Protein-Like 94	XLOC_000010	224897-227173	-0.115	0.022	-0.869										-0.796		-0.873			-0.865	3.732	3.159	1.395	1.130	-0.988							
III	Bradi2g05445	60S ribosomal protein	XLOC_000017	253480-263904	1.084	0.719	-1.443	-1.189	1.875	1.475	0.691	1.020	0.987	1.291	0.230	-0.038	1.224	1.190	1.016	1.379	1.018	1.137	1.309	1.675	0.778	0.898	0.961	1.085	1.760	1.925	0.683	0.778	0.354	0.516
II	Bradi1g13040	Cupin domain containing protein	XLOC_000011	270333-274431			-2.134	-1.988	-1.420	-1.712																								
I	Bradi1g13040	Cupin domain containing protein	XLOC_000012	298222-300988			9.975	9.757	13.069	13.084		-2.136	-0.977	-1.188	1.262	1.050	-2.153		-0.935		-1.844	-1.616		-2.142	-0.702	4.603	-1.913		0.394	-2.133	1.699	1.851		

Note: Gray shaded columns indicate no sequence reads detected in RNAseq experiments.

Supplementary Table 2: Distribution of *TaMs1* G329A variant within spring wheat germplasm.

Variety / line	Allele call at position 329	Type	Male Fertility Phenotype
Chris	G:G	Variety	Fertile
ms1d sterile segregant (*ms1d*/*ms1d*)	A:A	EMS mutant	Sterile
ms1d fertile segregant (ms*1d*/*Ms1*)	A:G	EMS mutant	Fertile
ms1d fertile segregant (*Ms1*/*Ms1*)	G:G	EMS mutant	Fertile
ms1e sterile segregant (*ms1e*/*ms1e*)	G:G	EMS mutant	Sterile
ms1e fertile segregant (*ms1e*/*Ms1*)	G:G	EMS mutant	Fertile
ms1e fertile segregant (*Ms1*/*Ms1*)	G:G	EMS mutant	Fertile
ms1f sterile segregant (*ms1f*/*ms1f*)	G:G	EMS mutant	Sterile
ms1f fertile segregant (*ms1f*/*Ms1*)	G:G	EMS mutant	Fertile
ms1f fertile segregant (*Ms1*/*Ms1*)	G:G	EMS mutant	Fertile
Cornerstone	G:G	Variety	Fertile
Cornerstone fertile segregant (*Ms1*/*Ms1*)	G:G	Deletion mutant	Fertile
Cornerstone sterile segregant (*ms1c*/*ms1c*)	No amplification	Deletion mutant	Sterile
Pastor	G:G	Variety	Fertile
RAC875	G:G	Variety	Fertile
AGT Katana	G:G	Variety	Fertile
AGT Scythe	G:G	Variety	Fertile
AGT Young	G:G	Variety	Fertile
Annuello	G:G	Variety	Fertile
Aroona	G:G	Variety	Fertile
Arrino	G:G	Variety	Fertile
Arrivato (*T. turgidum ssp. durum*)	G:G	Variety	Fertile
Axe	G:G	Variety	Fertile
Barham	G:G	Variety	Fertile
Batavia	G:G	Variety	Fertile
Baxter	G:G	Variety	Fertile
Binnu	G:G	Variety	Fertile
Bolac	G:G	Variety	Fertile

Bowie	G:G	Variety	Fertile
Braewood	G:G	Variety	Fertile
Brennan	G:G	Variety	Fertile
Brookton	G:G	Variety	Fertile
BT-Schomburgk	G:G	Variety	Fertile
Bullaring	G:G	Variety	Fertile
Bumper	G:G	Variety	Fertile
Cadoux	G:G	Variety	Fertile
Calingiri	G:G	Variety	Fertile
Caparoi (*T. turgidum ssp. durum*)	G:G	Variety	Fertile
Carinya	G:G	Variety	Fertile
Carnamah	G:G	Variety	Fertile
Cascades	G:G	Variety	Fertile
Chara	G:G	Variety	Fertile
Chinese Spring	G:G	Variety	Fertile
Clearfield Wht Jnz	G:G	Variety	Fertile
Clearfield Wht Stl	G:G	Variety	Fertile
Cobra	G:G	Variety	Fertile
Cook	G:G	Variety	Fertile
Corack	G:G	Variety	Fertile
Correll	G:G	Variety	Fertile
Cranbrook	G:G	Variety	Fertile
Cunningham	G:G	Variety	Fertile
Derrimut	G:G	Variety	Fertile
Diamondbird	G:G	Variety	Fertile
Drysdale	G:G	Variety	Fertile
Dundee	G:G	Variety	Fertile
EGA Bellaroi (*T. turgidum ssp. durum*)	G:G	Variety	Fertile
EGA Bonnie Rock	G:G	Variety	Fertile
EGA Bounty	G:G	Variety	Fertile
EGA Burke	G:G	Variety	Fertile

EGA Castle Rock	G:G	Variety	Fertile
EGA Eagle Rock	G:G	Variety	Fertile
EGA Eaglehawk	G:G	Variety	Fertile
EGA Gregory	G:G	Variety	Fertile
EGA Hume	G:G	Variety	Fertile
EGA Jitarning	G:G	Variety	Fertile
EGA Kidman	G:G	Variety	Fertile
EGA Stampede	G:G	Variety	Fertile
EGA Wedgetail	G:G	Variety	Fertile
EGA Wentworth	G:G	Variety	Fertile
EGA Wills	G:G	Variety	Fertile
EGA Wylie	G:G	Variety	Fertile
Einstein	G:G	Variety	Fertile
Ellison	G:G	Variety	Fertile
Elmore CL PLus	G:G	Variety	Fertile
Emu Rock	G:G	Variety	Fertile
Endure	G:G	Variety	Fertile
Espada	G:G	Variety	Fertile
Estoc	G:G	Variety	Fertile
Excalibur	G:G	Variety	Fertile
Fang	G:G	Variety	Fertile
Festiguay	G:G	Variety	Fertile
Forrest	G:G	Variety	Fertile
Fortune	G:G	Variety	Fertile
Frame	G:G	Variety	Fertile
Gabo	G:G	Variety	Fertile
GBA Combat	G:G	Variety	Fertile
GBA Hunter	G:G	Variety	Fertile
GBA Ruby	G:G	Variety	Fertile
GBA Sapphire	G:G	Variety	Fertile
Giles	G:G	Variety	Fertile

Gladius	G:G	Variety	Fertile
Glover	G:G	Variety	Fertile
Grenade CL Plus	G:G	Variety	Fertile
H45	G:G	Variety	Fertile
H46	G:G	Variety	Fertile
Halberd	G:G	Variety	Fertile
Harrismith	G:G	Variety	Fertile
Hartog	G:G	Variety	Fertile
Hyperno (*T. turgidum ssp. durum*)	G:G	Variety	Fertile
Impala	G:G	Variety	Fertile
Impose CL Plus	G:G	Variety	Fertile
Jandaroi (*T. turgidum ssp. durum*)	G:G	Variety	Fertile
Janz	G:G	Variety	Fertile
Justica CL Plus	G:G	Variety	Fertile
Kalka (*T. turgidum ssp. durum*)	G:G	Variety	Fertile
Kelalac	G:G	Variety	Fertile
Kennedy	G:G	Variety	Fertile
King Rock	G:G	Variety	Fertile
Kord CL Plus	G:G	Variety	Fertile
Krichauff	G:G	Variety	Fertile
Kukri	G:G	Variety	Fertile
Kunjin	G:G	Variety	Fertile
Lancer	G:G	Variety	Fertile
Lang	G:G	Variety	Fertile
Lincoln	G:G	Variety	Fertile
Livingstone	G:G	Variety	Fertile
Longreach Beaufort	G:G	Variety	Fertile
Longreach Catalina	G:G	Variety	Fertile
LongReach Cobra	G:G	Variety	Fertile
LongReach Crusader	G:G	Variety	Fertile
Longreach Dakota	G:G	Variety	Fertile

LongReach Dart	G:G	Variety	Fertile
LongReach Envoy	G:G	Variety	Fertile
LongReach Gauntlet	G:G	Variety	Fertile
LongReach Gazelle	G:G	Variety	Fertile
Longreach Guardian	G:G	Variety	Fertile
LongReach Impala	G:G	Variety	Fertile
LongReach Lincoln	G:G	Variety	Fertile
LongReach Orion	G:G	Variety	Fertile
LongReach Phantom	G:G	Variety	Fertile
LongReach Scout	G:G	Variety	Fertile
LongReach Spitfire	G:G	Variety	Fertile
Lorikeet	G:G	Variety	Fertile
Mace	G:G	Variety	Fertile
Machete	G:G	Variety	Fertile
Mackellar	G:G	Variety	Fertile
Magenta	G:G	Variety	Fertile
Mansfield	G:G	Variety	Fertile
Maringa	G:G	Variety	Fertile
Marombi	G:G	Variety	Fertile
Meering	G:G	Variety	Fertile
Merinda	G:G	Variety	Fertile
Merlin	G:G	Variety	Fertile
Molineux	G:G	Variety	Fertile
Naparoo	G:G	Variety	Fertile
Orion	G:G	Variety	Fertile
Peake	G:G	Variety	Fertile
Pelsart	G:G	Variety	Fertile
Petrel	G:G	Variety	Fertile
Preston	G:G	Variety	Fertile
Pugsley	G:G	Variety	Fertile
QAL2000	G:G	Variety	Fertile

QALBIS	G:G	Variety	Fertile
Raven	G:G	Variety	Fertile
Rees	G:G	Variety	Fertile
Rosella	G:G	Variety	Fertile
Rudd	G:G	Variety	Fertile
Saintly (*T. turgidum ssp. durum*)	G:G	Variety	Fertile
Scout	G:G	Variety	Fertile
Sentinel	G:G	Variety	Fertile
Seri 82	G:G	Variety	Fertile
Shield	G:G	Variety	Fertile
Silverstar	G:G	Variety	Fertile
Spear	G:G	Variety	Fertile
Spitfire	G:G	Variety	Fertile
SQP Revenue	G:G	Variety	Fertile
Stiletto	G:G	Variety	Fertile
Strzelecki	G:G	Variety	Fertile
Sunbri	G:G	Variety	Fertile
Sunco	G:G	Variety	Fertile
Sunelg	G:G	Variety	Fertile
Sunguard	G:G	Variety	Fertile
Sunlin	G:G	Variety	Fertile
Sunsoft 98	G:G	Variety	Fertile
Sunstate	G:G	Variety	Fertile
Suntop	G:G	Variety	Fertile
Sunvale	G:G	Variety	Fertile
Sunvex	G:G	Variety	Fertile
Sunzell	G:G	Variety	Fertile
Tamaroi (*T. turgidum ssp. durum*)	G:G	Variety	Fertile
Tammarin Rock	G:G	Variety	Fertile
Tammin	G:G	Variety	Fertile
Tasman	G:G	Variety	Fertile

Tennant	G:G	Variety	Fertile
Thatcher	G:G	Variety	Fertile
Tjilkuri (*T. turgidum ssp. durum*)	G:G	Variety	Fertile
Trident	G:G	Variety	Fertile
UAD0951096 (*T. turgidum ssp. durum*)	G:G	Variety	Fertile
Ventura	G:G	Variety	Fertile
Waagan	G:G	Variety	Fertile
Wallup	G:G	Variety	Fertile
Wedin	G:G	Variety	Fertile
Westonia	G:G	Variety	Fertile
Whistler	G:G	Variety	Fertile
WID802 (*T. turgidum ssp. durum*)	G:G	Variety	Fertile
Wollaroi (*T. turgidum ssp. durum*)	G:G	Variety	Fertile
Worrakatta	G:G	Variety	Fertile
Wyalkatchem	G:G	Variety	Fertile
Wylah	G:G	Variety	Fertile
Yallaroi (*T. turgidum ssp. durum*)	G:G	Variety	Fertile
Yandanooka	G:G	Variety	Fertile
Yawa	G:G	Variety	Fertile
Yenda	G:G	Variety	Fertile
Yitpi	G:G	Variety	Fertile
Zebu	G:G	Variety	Fertile
Zippy	G:G	Variety	Fertile
Zulu (*T. turgidum ssp. durum*)	G:G	Variety	Fertile

Supplementary Table 3: Fertility in T_0 wheat plants containing a *TaMs1* complementation T-DNA insertion.

**T-DNA Insertion Event**	***ms1d* Genotype**	**T-DNA Copy Number**	**Male Fertility Phenotype**
Event-1	*ms1d/ms1d*	1	Fertile
Event-2	*ms1d/ms1d*	1	Fertile
Event-3	*ms1d/ms1d*	2	Fertile
Event-4	*ms1d/ms1d*	2	Fertile
Event-5	*ms1d/ms1d*	3	Fertile
Event-6	*ms1d/ms1d*	4	Fertile
Event-7	*Ms1/ms1d*	1	Fertile
Event-8	*Ms1/ms1d*	1	Fertile
Event-9	*Ms1/ms1d*	1	Fertile
Event-10	*Ms1/ms1d*	1	Fertile
Event-11	*Ms1/ms1d*	1	Fertile
No T-DNA	*ms1d/ms1d*	0	Sterile
No T-DNA	*ms1d/ms1d*	0	Sterile

Supplementary Table 4: Fertility of T_1 plants with or without a *TaMs1* complementation T-DNA insertion.

T_0 Event	T_1 Plant	*ms1d* genotype	T-DNA Copy Number	Male Fertility Phenotype
Event-1	Plant 1	homozygous	1	Fertile
Event-1	Plant 2	homozygous	1	Fertile
Event-1	Plant 3	homozygous	1	Fertile
Event-1	Plant 4	homozygous	1	Fertile
Event-1	Plant 5	homozygous	2	Fertile
Event-1	Plant 6	homozygous	2	Fertile
Event-1	Plant 7	homozygous	2	Fertile
Event-1	Plant 8	homozygous	0	Sterile
Event-1	Plant 9	homozygous	0	Sterile
Event-7	Plant 1	homozygous	1	Fertile
Event-7	Plant 2	homozygous	1	Fertile
Event-7	Plant 3	homozygous	1	Fertile
Event-7	Plant 4	homozygous	2	Fertile
Event-7	Plant 5	homozygous	2	Fertile
Event-7	Plant 6	homozygous	2	Fertile
Event-7	Plant 7	homozygous	0	Sterile
Event-7	Plant 8	homozygous	0	Sterile

Supplementary Table 5: Primers used for high resolution melting (HRM) marker analysis

Marker	Forward Primer (5’ to 3’)	Reverse Primer (5’ to 3’)	Amplicon Sequence
x21056	ATGATGGATGGACGGATGAT	AGTTTTGCTGCGTTTGGACT	ATGATGGATGGACGGATGATGCTCTGTGGTGCCYTGTATTTATATTTGTTGAGKTCT<br>TTGATACAGGATGATATGTCTCCAAYGTATCTATAATTTTTTGTTGTTCCATGTTGTTA<br>TATTATCATTTTGRATGTTTTACAATCATTTTATAATCATTCTATATCATTTTTTGTWA<br>CTAACCTATTKACATAGTGCCAAGTGCTAGTTGTTGTTTTCTGCTTGTTTTTTACATCG<br>CAGGAAATCAATACCAAAYGGAGTCCAAACGCAGCAAAACT
x27140346<br>wsnp_Ex_c18318_27140346	TACGTGGGTGGAAAATAAATTCTG	GGATTTGAATTACCGGATAGCG	TACGTGGGTGGAAAATAAATTCTGCCTTCTTTCCAGCCCGCGAATACCCGACCAAAC<br>CTTTGCTCTATACGAAGCTGTTGCAGAAAATTTTCAGGTAAGAAACGAGGCCAAGA<br>ATGTGATTGCAATAGCTGCGCAAGATGGGCAATTCTCTTCTGCTTAATTAATCGATG<br>AGGCAAATCTTTTGCCTCCGTTTCAAAAAAATATAGCTGCGCAAGTTGCTTTAAGGG<br>GCATATGATATGGCTTACCTGATCTTTTTCCAACAATTGTTTCCAATTGTGCATCTCAA<br>CCAAAGCCTTCACAGAGCGRTAGGAAAGCGCTATCCGGTAATTCAAATCC
x12360198<br>wsnp_Ku_c7153_12360198	CCAACTTGCTTGGTGTTCCT	CAGGAAATCCCAGCTCACC	TATATGAATTAGGCTTTGCGGACCGGAAGTACAACGCCAACTTGCTTGGTGTTCCTG<br>CACTGCTGCTTGAGACCTTGATGTGCAAAGTTATGAACATATCRGCGTAACTAGGGA<br>AAGTGAATATGTATAATACTAGCAAACAAGTTAGATTAGGCTAAATCGCGYTATTTT<br>CCGGTGAGCTGGGATTTCCTGGCCAGT
xBF292015	TTAATAGTGTTCCGCCCTGC	AAAACCACGACAAGTATTTTGGA	TTAATAGTGTTCCGCCCTGCGTGGCACAGACCGTAACGAATGATTGTACGAACGTTT<br>TTCACTAATCATTACATAGGTGTGGTTTAAGAAAAAATCAACCACCTTAACGCCGTA<br>ATCCCCTCTACAGAATAACCACACAAGTCATGAAAAGGAACTATTAACAAAAATAGA<br>TGATAAAATAAATCTCAAATCCAAATTCATAAAGCACCTCTAGTCTATAAACWCGAC<br>MAATCTAGCTATGTTTAGTACTTCMTCCGTTCCAAAATACTTGTCGTGGTTTT

Supplementary Table 6: Primers used for KASPar analysis. Tag sequences in primers are undelined.

Marker	Primer Allele X (5' to 3')	Primer Allele Y (5' to 3')	Common Primer (5' to 3')	Allele X	Allele Y	Sequence
ET0488<br>007-0060.1	GAAGGTGACCAAGTTCATGCTGGTTTTGGGTTTACTTTGAGTCATAGA	GAAGGTCGGAGTCAACGGATTGTTTTGGGTTTACTTTGAGTCATAGG	AGCAACCCCCTCTTTATAGTATGG	Gladius	Chris	GTTTTGGGTTTACTTTGAGTCATAG[R]AGAAGCCATACTATAAAGAGGGGGTTGCT
ET0489<br>007-0061.1	GAAGGTGACCAAGTTCATGCTTTTCCCTTGTCAAGTCATTGCC	GAAGGTCGGAGTCAACGGATTCTTTCCCTTGTCAAGTCATTGCA	ACTTGATTGTACTTTTTGGTTAGCAAC	Gladius	Chris	ACTTGATTGTACTTTTTGGTTAGCAACAAT[K]GCAATGACTTGACAAGGGAAA
ET0490<br>007-0062.1	GAAGGTGACCAAGTTCATGCTGCCTCCTCGACCTTTTCCTTG	GAAGGTCGGAGTCAACGGATTGCCTCCTCGACCTTTTCCTTC	CTCGCCTTGGCTATAGGGTCC	Chris	Gladius	CTCGCCTTGGCTATAGGGTCCTCTGTGAG[S]AAGGAAAAGGTCGAGGAGGC
ET0491<br>007-0063.1	GAAGGTGACCAAGTTCATGCTCAACACATCCGAACATGCTTCT	GAAGGTCGGAGTCAACGGATTAACACATCCGAACATGCTTCC	CAACGCTATCCCTTAAAACGG	Chris	Gladius	CAACGCTATCCCTTAAAACGGATACACTATCCATCCG[R]GAAGCATGTTCGGATGTGTTG
007-0033.1	GAAGGTGACCAAGTTCATGCTGGAAAGTCCAAAGGTAATTACCCAG	GAAGGTCGGAGTCAACGGATTGGAAAGTCCAAAGGTAATTACCCAT	ATCACATTGCACAAGTTAATAGTCCGGTA	Gladius	Chris	ATCACATTGCACAAGTTAATAGTCCGGTA[M]TGGGTAATTACCTTTGGACTTTCCA
007-0046.1	GAAGGTGACCAAGTTCATGCTGTTTCTGCTGCTTGCTCTGCTTATAA	GAAGGTCGGAGTCAACGGATTGTTTCTGCTGCTTGCTCTGCTTATAT	CATGAACAGACCATTCGCACATATCATTA	Gladius	Chris	GTTTCTGCTGCTTGCTCTGCTTATA[W]AATGATAATGATATGTGCGAATGGTCTGTTCATG
007-0021.1	GAAGGTGACCAAGTTCATGCTCTATAGACAAAATTGTTAAGATCTGACGT	GAAGGTCGGAGTCAACGGATTCTATAGACAAAATTGTTAAGATCTGACGC	YAGCAGCGACGGTGTGGAGGTT	Chris	Gladius	CTATAGACAAAATTGTTAAGATCTGACG[Y]CGCTCCTCCTGCCAACCTCCACACCGTCGCTGCTR

007-0008.1	GAAGGTGACCAAGTTCATGCTCAGTGGTTCATCTTAACCAGAGGAA	GAAGGTCGGAGTCAACGGATTAGTGGTTCATCTTAACCAGAGGAC	GCGTGGTCGCGGAGAACAATTAATT	Chris	Gladius	TCAGTGGTTCATCTTAACCAGAGGA[M]TCGCAAACACCCAATTAATTGTTCTCCGCGACCACGC
007-0009.1	GAAGGTGACCAAGTTCATGCTGGTTTTCCCTAGTAGTGTGAGGA	GAAGGTCGGAGTCAACGGATTGGTTTTCCCTAGTAGTGTGAGGG	GAGCGAGTGGGATGAGGCGTT	Chris	Gladius	GGTTTTCCCTAGTAGTGTGAGG[R]GAACGCCTCATCCCACTCGCTC
007-0029.1	GAAGGTGACCAAGTTCATGCTCCTCCTAGATGGCACCACACATA	GAAGGTCGGAGTCAACGGATTCTCCTAGATGGCACCACACATC	CAAGGAAATTAAGTGTTCATTCTTGGTGTA	Gladius	Chris	GGAAATTAAGTGTTCATTCTTGGTGTATTTCAAAGAAAGAAAAAA[K]ATGTGTGGTGCCATCTAGGAGG
007-0017.1	GAAGGTGACCAAGTTCATGCTCCTGTCCCTGATCACCCCG	GAAGGTCGGAGTCAACGGATTCCCTGTCCCTGATCACCCCA	GATTGGGCGCGGGCAAATGGAT	Chris	Gladius	GATTGGGCGCGGGCAAATGGATGGGG[Y]GGGGTGATCAGGGACAGG
007-0042.1	GAAGGTGACCAAGTTCATGCTGTCAAGCCCAAGCCCGGCT	GAAGGTCGGAGTCAACGGATTCAAGCCCAAGCCCGGCC	GGCCTAAAATTTGAGCCCGAAGGTT	Gladius	Chris	GGCCTAAAATTTGAGCCCGAAGGTTG[R]GCCGGGCTTGGGCTTGAC
007-0091.1	GAAGGTGACCAAGTTCATGCTGGAGGAGGCGGACAACGTAC	GAAGGTCGGAGTCAACGGATTGGAGGAGGCGGACAACGTAT	CTCGCCGCCGCCTGCGAA	*Ms1d*	*ms1d*	CTCGCCGCCGCCTGCGAAG[R]TACGTTGTCCGCCTCCTCC

Supplementary Table 7: Primers used for sequencing *TaMs1*, *TaMs1* and homeologue qRT-PCR and expression analysis

Gene	Purpose	Forward Primer (5' to 3')	Reverse Primer (5' to 3')
TaMs1 (coding region)	*TaMs1* amplification	GCATTCCATTCCGCCACCG	TTAGGTTTCCTCGTGTTCTAAAGAGC
TaMs1_B	qRT-PCR	CCTCTACATCATCCTCTGAGTCGC	GTACGAGCGGACAGAAACGATAG
TaMs1_A	qRT-PCR	CCTCTACATCATCCTCTGAGTCGC	TGAACATACTGCTGCTACCAGACACTA
TaMs1_D	qRT-PCR	CCTCTACATCATCCTCTGAGTGGC	TCCATACTCCTGCCAACGACAG
*TaEFA 2*2* Elongation Factor alpha	qRT-PCR	CAGATTGGCAACGGCTACG	CGTAACCCAAAATGCCCTTG
*TaGAPdH 2*2* Glyceraldehyde-3-phosphate dehydrogenase	qRT-PCR	TTCAACATCATTCCAAGCAGCA	CGGACAGCAAAACGACCAAG
*TaCyclophilin 2*2* Cyclophilin	qRT-PCR	CAAGCCGCTGCACTACAAGG	AGGGGACGGTGCAGATGAA
